# π‑Conjugated Triazine–Benzotrithiophene COF Networks Integrated with Carbon Nanotubes and Reduced Graphene Oxide in Cellulose Films for High-Performance Supercapacitors

**DOI:** 10.1021/acspolymersau.5c00111

**Published:** 2025-10-20

**Authors:** Yi-Yun Chen, Mahmoud Younis, Pei-Cih Hu, Peng-Yao Chen, Cheng-Yeh Hsin, Hongta Yang, Bo-Tau Liu, Rong-Ho Lee

**Affiliations:** 1 Department of Chemical Engineering, 34916 National Chung Hsing University, Taichung 402, Taiwan; 2 Chemistry Department, Faculty of Science, New Valley University, El-Kharja 72511, Egypt; 3 Department of Chemical and Materials Engineering, 34883 National Yunlin University of Science and Technology, Yunlin 64002, Taiwan; 4 Department of Chemical Engineering and Materials Science, Yuan Ze University, Taoyuan City 320, Taiwan

**Keywords:** triazine, benzotrithiophenes, covalent organic framework, capacitance, supercapacitor

## Abstract

This study presents the synthesis of a covalent organic framework (TPBT-COF) via the Schiff-base reaction and its integration with carbon nanotubes (CNTs) and reduced graphene oxide (rGO) through in situ polymerization, yielding TPBT@CNT and TPBT@rGO composites. To develop environmentally friendly electrode materials, the TPBT@CNT and TPBT@rGO composites were blended with regenerated cellulose (RC), forming TPBT@CNT/CNT/RC and TPBT@rGO/rGO/RC films. The TPBT@CNT/CNT/RC film-based electrode exhibited superior capacitive performance due to its uniform composition, achieving a specific capacitance of 1288.26 F/g at 0.5 A/g. In contrast, the TPBT@rGO/rGO/RC film-based electrode showed a lower capacitance of 398.75 F/g at 0.5 A/g, attributed to the uneven material distribution. Both composite film-based electrodes demonstrated excellent cycling stability, retaining 85.87 and 81.82% of their initial capacitance after 10,000 cycles, respectively. In a symmetric device configuration, the TPBT@CNT-50/CNT/RC (35/35/30, w/w) electrode achieved a specific capacitance of 84.32 F/g at 1 A/g, with a maximum energy density of 11.71 Wh/kg and a power density of 312.5 W/kg, while maintaining 77% of its initial capacitance after 10,000 cycles. These findings underscore the potential of TPBT-COF-based composites as sustainable, high-performance electrode materials for energy storage applications.

## Graphical abstract

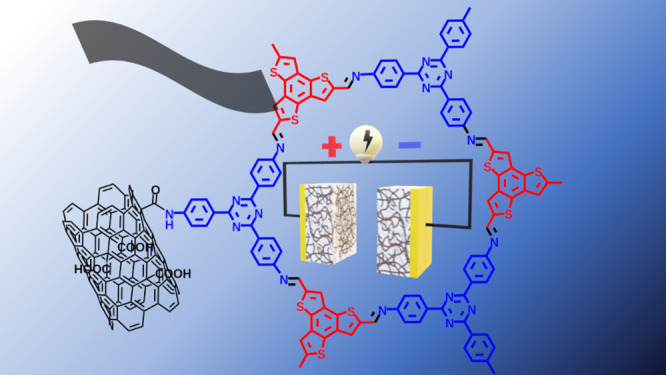

## 1 Introduction

The rapid depletion of fossil fuel reserves, coupled with their severe environmental consequences, has heightened the urgent need for efficient and sustainable technologies for clean energy conversion and storage.^1^ In response to this global challenge, the demand for innovative energy storage technologies has surged, driving the development of novel energy storage devices. Supercapacitors, also known as electrochemical capacitors (ECs), have emerged as a cutting-edge energy storage technology, attracting considerable scientific attention in recent years.^2^ The performance of supercapacitors is largely determined by their electrode materials.^3^ To date, a wide range of electrode materials has been extensively explored, including activated carbon (AC),^4^ conducting polymers,^5^ redox-active polymers,^6^ metal–organic frameworks (MOFs),^7^ and covalent organic frameworks (COFs).^8–11^

COFs have gained significant attention as promising candidates for supercapacitor applications due to their tunable porosity, exceptionally large surface area, high crystallinity, and structural versatility.^12,13^ Two-dimensional (2D) COFs, featuring π-conjugated frameworks and abundant open nanopores, provide an ideal platform for charge transport and energy storage while maximizing the formation of electrochemical double layers (EDLs).^14^ Additionally, the presence of electrochemically active functional groups within these materials contributes to pseudocapacitive effects, further enhancing their overall performance. These intrinsic properties make 2D COFs outstanding electrode materials for supercapacitor applications.^12,15^ The incorporation of heterostructures containing lone-pair electrons into the carbon skeleton can modulate the electronic structure, thereby enhancing the supercapacitive properties.^16,17^ The multiple nitrogen groups in triazine structures make them particularly appealing for the development of COFs with superior electrochemical properties. Incorporating triazine units into COFs allows for tuning redox properties and improving charge storage capabilities, positioning them as promising candidates for next-generation energy storage materials.^10,18–22^

Benzotrithiophenes (BTT) represent promising supramolecular building units owing to their distinctive planar molecular architecture, extended π-conjugated framework, and electron-rich heteroaromatic character. These intrinsic properties promote enhanced intermolecular π-π stacking interactions, leading to the formation of supramolecular assemblies and facilitating efficient intermolecular charge transport mechanisms.^23^ The extended thiophene/benzene-fused π-conjugated system inherent to BTT structures significantly enhances the electronic conductivity of the resultant COF materials. The star-shaped conjugated geometry of BTT units substantially reinforces π-π stacking interactions within COF architectures. COFs incorporating planar π-extended monomers, particularly thiophene derivatives, demonstrate enhanced π-conjugation and strengthened π-π interactions, thereby optimizing charge transfer efficiency.^24^ Notably, 2D polymer systems containing highly planar, polarizable, and electron-rich thiophene moieties typically exhibit superior charge carrier mobility along the polymeric backbone. Under high-voltage electrochemical conditions, the electron-rich thiophene conjugated framework undergoes controlled electron depletion, creating favorable conditions for anion accommodation while simultaneously improving discharge voltage stability and enhancing specific capacity values.^25^ The star-shaped planar configuration of BTT further amplifies π-π stacking interactions within COF structures, promoting efficient intermolecular charge transfer via optimized hopping mechanisms.^26^ Additionally, the electron-rich nature of BTT units provides supplementary electrochemical capacity through reversible anion coordination under elevated voltage conditions. Ringk et al. reported the preparation of BTT and 3,4-ethylenedioxythiophene (EDOT)-based copolymers via the electrochemical polymerization method. The resulting BTT/EDOT copolymers exhibited a high areal capacitance of 443.8 mF cm^–^^2^ along with good cycling stability, retaining 89% of their capacitance after 1,000 cycles.^27^

However, the inherently low electrical conductivity of COFs poses a significant challenge to their practical implementation in energy storage devices. To overcome this limitation, various strategies have been explored, including the integration of COFs with carbon nanotubes (CNTs) and graphene nanomaterials^9,28,29^ and the incorporation of conductive polymers into the COF backbone.^30–32^ The integration of CNTs with COFs to form CNT/COF composites represents a particularly promising approach, especially for energy storage and conversion applications. These hybrid materials effectively harness the exceptional electrical conductivity and mechanical robustness of CNTs, while simultaneously leveraging the high surface area, customizable pore architecture, and versatile chemical functionality of COFs. Numerous investigations have explored the potential of CNT/COF composites in various energy-related applications, including supercapacitors, batteries, and electrocatalysis.^12,33^ Similarly, graphene, alongside CNTs, has attracted considerable scientific and technological interest, with multiple studies reporting the development of graphene/COF composites for energy conversion and storage applications.^34^

Yang et al. reported the synthesis of triazine-based two-dimensional COFs via Schiff base condensation reactions and their subsequent integration with carbon nanotube fibers (f-CNFs), yielding TPTP-COF@f-CNF and TPDA-COF@f-CNF nanocomposites. Electrochemical characterization revealed that electrodes fabricated from TPTP-COF and TPDA-COF exhibited notable specific capacitances of 577.4 F g^–^^1^ and 640.4 F g^–^^1^, respectively, when evaluated at a scan rate of 5 mV s^–^^1^. Solid-state supercapacitor devices constructed from these materials achieved maximum specific capacitances of 56.4 F g^–^^1^ and 70.6 F g^–^^1^, respectively, while demonstrating exceptional electrochemical stability during extended cycling. The TPTP-COF-based device retained 78.60% of its initial capacitance after 10,000 charge–discharge cycles, while the TPDA-COF-based device maintained 81.54% capacitance retention.^35^ In a parallel investigation, Liu et al. synthesized triazine-COF@CNT composites through the incorporation of carboxylated multiwalled CNTs via a facile one-pot methodology, wherein two-dimensional TFA-COFs were grown in situ on the surface of functionalized CNTs. Among the prepared composites, CNT@TFA-COF-3 exhibited superior crystallinity, well-defined porosity, remarkable stability, and a specific surface area of 1034 m^2^ g^–^^1^. When evaluated as a capacitive electrode material, the CNT@TFA-COF composite demonstrated enhanced electrochemical performance. Notably, the specific capacitance of CNT@TFA-COF-3 (338 F g^–^^1^ at 1.0 A g^–^^1^) was approximately 8.5, 4.9, and 7.5 times greater than that of pristine TFA-COFs, CNTs, and physically mixed CNT/TFA-COF complexes, respectively. Furthermore, the CNT@TFA-COF-3 supercapacitor exhibited exceptional cycling stability and rate capability, maintaining performance even after 7000 charge–discharge cycles.^33^ Ibrahim et al. investigated both ex-situ and in situ (one-pot) synthetic approaches for triazine COF/graphene oxide (GO) nanocomposites. These materials were subsequently converted to nitrogen-doped carbon (N-doped C)/reduced GO (rGO) via carbonization. When evaluated as electrode materials for supercapacitor applications, the N-doped C/rGO synthesized via the in situ method exhibited superior electrochemical performance compared to materials prepared via ex-situ procedures. The in situ synthesized N-doped C/rGO delivered a specific capacitance of 234 F g^–^^1^ at a current density of 0.8 A g^–^^1^. Symmetric supercapacitor devices fabricated using these materials successfully powered white light-emitting diode (LED) lamps. Notably, devices employing N-doped C/rGO in situ electrodes demonstrated a high specific energy of 14.6 Wh kg^–^^1^ and specific power of 400 W kg^–^^1^, with only 14% capacitance degradation after 3500 cycles.^36^ A tetraphenylethylene-based COF (TTPE-COF) with a nearly pure carbon framework was chemically grafted onto aniline-functionalized graphene oxide (a-GO) to form a-rGO@TTPE-COF composites. Among them, the a-rGO@TTPE-COF-3 composite demonstrated a remarkable improvement in electrochemical performance as a capacitive electrode material. At a current density of 1.0 A g^–^^1^, a-rGO@TTPE-COF-3 exhibited a specific capacitance of 139 F g^–^^1^, representing a 20% increase compared with a-rGO (116 F g^–^^1^) and far exceeding that of pristine TTPE-COF (5.0 F g^–^^1^).^37^

In this work, we present the synthesis of a covalent organic framework (TPBT-COF) via Schiff-base reaction between 2,4,6-tris­(4-aminophenyl)-1,3,5-triazine (TPT-NH_2_) and benzo­[1,2-b:3,4-b’:5,6-b’’]­trithiophene-2,5,8-tricarboxaldehyde (BTT-CHO) monomers and its strategic integration with CNTs and GO through in situ polymerization to yield TPBT@CNT and TPBT@rGO composites. This approach effectively addresses the inherent aggregation issues of COFs while simultaneously leveraging their advantageous properties, including high surface area and crystallinity, in conjunction with the exceptional conductivity of carbon nanomaterials. We further demonstrate the fabrication of environmentally friendly electrode materials by blending these composites with regeneration cellulose (RC) to produce TPBT@CNT/CNT/RC and TPBT@rGO/rGO/RC films. The incorporation of CNT and rGO into the composite films enhanced both the conductivity and the electrochemical double-layer (EDL) capacitance. These cellulose-based composite films were successfully utilized in the development of flexible supercapacitors, highlighting their potential for sustainable energy storage applications.^38^

## 2 Experiment

### 2.1 Materials

4-Aminobenzonitrile (C_7_H_6_N_2_, Matrix, 98%), ascorbic acid (Vetec, 99%), n-butanol (C_4_H_1__0_O, Sigma-Aldrich, 99.8% and J.T. Baker, 99.4%), BTT-CHO (C_1__5_H_6_O_3_S_3_, Extension, 97+%), 1,2-dichlorobenzene (o-DCB, C_6_H_4_Cl_2_, Alfa, 98%, spectrophotometric grade), graphene oxide (GO, Taiwan Carbon Materials, 99%), mesitylene (C_9_H_1__2_, Alfa, 98+%), multiwalled carbon nanotubes (MWF-CNTs, Taiwan Carbon Materials), trifluoromethanesulfonic acid (CF_3_SO_3_H, Alfa, 98+%), urea (CON_2_H_4_, J.T. Baker, 99%), and thiourea (SC­(NH_2_)_2_, Showa Chemical Industry, 98%) were used as received without further purification. The *f*-CNTs and RC were prepared according to the previous literature.^38^ The synthesis of the TPT-3NH2 and TPBT-COF have been reported in the previous literature.^39^

### 2.2 Synthesis of TPBT-COF@CNT and TPBT-COF@rGO Composites

The synthesis routes of the TPBT-COF@f-CNT and TPBT-COF@GO composites are illustrated in Schemes 1. Both composites were prepared using a similar solvothermal approach with identical reaction conditions but different carbon nanomaterials. A 25 mL single-neck round-bottom flask was charged with TPT-3NH_2_ (62.02 mg) and BTT-CHO (57.82 mg), along with the respective carbon nanomaterial: f-CNT at varying weight loadings (30, 50, or 70 wt % relative to total monomer weight) or pretreated GO (at 30, 40, or 50 wt % relative to total monomer weight). The mixture was combined with 5 mL of a solvent blend consisting of n-butanol, 1,2-dichlorobenzene, and acetic acid. The reaction mixture was heated to 120 °C and maintained at this temperature for 3 days. After cooling to room temperature, the products were collected via vacuum filtration and purified through sequential washings with methanol, acetone, tetrahydrofuran, and dimethyl sulfoxide, followed by drying in a vacuum oven at 120 °C, yielding blue-black products with consistent 89% yields TPBT@f-CNT-30 (152.37 mg), TPBT@f-CNT-50 (213.52 mg), and TPBT@f-CNT-70 (279.63 mg). The TPBT@GO composites were obtained as dark green solids in 82% yield: TPBT@GO-30 (140.38 mg), TPBT@GO-40 (163.78 mg), and TPBT@GO-50 (196.55 mg).

**Scheme 1:**
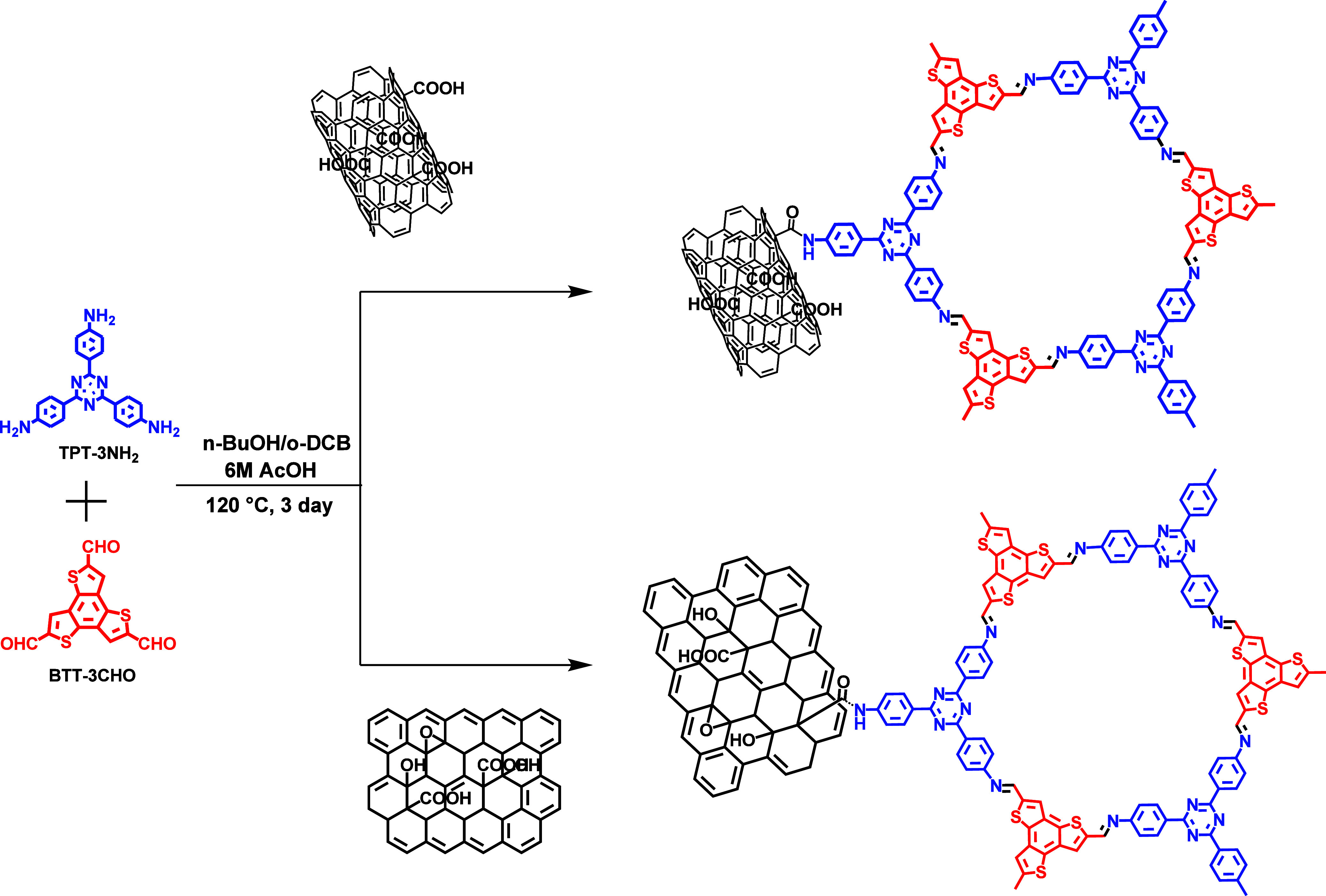
Synthesis Route of the TPBT@f-CNT and TPBT@rGO Nanocomposites

### 2.3 Reduction of TPBT-COF Composites

Both TPBT@f-CNT and TPBT@GO composites were subjected to reduction using a standardized ascorbic acid protocol. Ascorbic acid (4.405 g) was dissolved in deionized water (500 mL) with stirring until a clear, colorless solution was obtained. The pH was adjusted to 10.0 using 0.1 M NaOH. The respective composite powders (TPBT@f-CNT or TPBT@GO at various loadings) were dispersed in this basic solution, and the reaction mixture was heated to 90 °C and maintained for 2 h with continuous stirring. After cooling to room temperature, the products were collected by vacuum filtration and washed thoroughly with deionized water to remove residual ascorbic acid. The materials were dried in a vacuum oven at 120 °C for 4 h, yielding the reduced composites: TPBT@CNT-30, TPBT@CNT-50, TPBT@CNT-70, and TPBT@rGO-30, TPBT@rGO-40, TPBT@rGO-50, respectively.

### 2.4 Instrumentation

Fourier transform infrared (FTIR) spectra was recorded using a HORIBA FT-720 FTIR spectrometer. The thermal decomposition temperatures (*T*_d_; temperature at which weight loss reached 5%) of the TPBT@CNT and TPBT@rGO composites were determined through thermogravimetric analysis (TGA, PerkinElmer Pyrisl). TGA analysis was conducted under a N_2_ atmosphere at a heating rate of 10 ◦C min^– 1^. The morphologies of the TPBT@CNT and TPBT@rGO composites were investigated using field emission scanning electron microscopy (FESEM, JSM 7401F; JEOL, Japan) and high-resolution transmission electron microscopy (HRTEM, JEOL JEM-1400). The crystalline structures of the TPBT@CNT and TPBT@rGO composites were examined using X-ray diffractometry (XRD, Rigaku RINT 2000, Tokyo, Japan) with Ni-filtered Cu Kα radiation. The chemical compositions of the TPBT@CNT and TPBT@rGO composites were measured using X-ray photoelectron spectroscopy (XPS, ESCALAB 250Xi, Thermo Fisher, USA). The surface areas and pore characteristics were determined using a surface area and porosity analyzer (ASAP 2010, Micromeritics, USA). The Brunauer–Emmett–Teller (BET) and the Barret–Joyner–Halenda (BJH) methods were used to estimate the specific surface areas and pore size distributions, respectively, of the TPBT@CNT and TPBT@rGO composites.

### 2.5 Electrochemical Measurements

#### 2.5.1 Preparation of TPBT Composite Electrodes

The TPBT@CNT/f-CNT/RC and TPBT@rGO/rGO/RC composite electrodes were prepared using an identical procedure with different carbon nanomaterials. Purified RC (30 mg) was dissolved in the prepared RC solution (1.5 mL) in a 50 mL beaker under ice-bath conditions with stirring. Simultaneously, the respective TPBT composite (TPBT@CNT or TPBT@rGO, 35 mg) and corresponding carbon nanomaterial (f-CNT or rGO, 35 mg) were dispersed in deionized water (20 mL) in a 30 mL beaker and sonicated for 2 h. The dispersed material was slowly added to the cellulose solution and stirred for 3 h. The resulting mixture was vacuum filtered, and the collected film was dried at 60 °C for 24 h. The dried composite films were cut into 1 cm × 1 cm pieces to obtain the final TPBT@CNT/f-CNT/RC and TPBT@rGO/rGO/RC electrode films.

#### 2.5.2 Electrochemical Properties of the Electrodes

The TPBT@CNT/CNT/RC and TPBT@rGO/rGO/RC composite films–based electrodes were placed in a three-electrode system containing 1 M H_2_SO_4_, with a Pt electrode as the counter electrode and a Hg/Hg_2_Cl_2_ electrode (SCE; Eo = 0.2412 V) as the reference electrode. A symmetric supercapacitor device was constructed from two TPBT-COF composite film-based electrodes and a filter paper separator soaked in 1 M H_2_SO_4_ (electrolyte). Using a CHI6273D electrochemical workstation (CH Instruments, USA), the electrochemical characteristics of the TPBT-COF composite film–based electrodes in a three-electrode configuration were investigated through cyclic voltammetry (CV) and the galvanostatic charge/discharge (GCD) method. The following eq 1 was used to calculate the gravimetric capacitance (*C*_m_, F g^–1^) of the composite electrodes.^36^

$$C_{m}=\frac{I\Delta t}{m\times \Delta V}$$ 1

where I, Δ*t*, m, and Δ*V* denote the discharge current (A), discharge time (s), weight of the freestanding electrode (g), and the potential window (V), respectively.^36^

The gravimetric capacitance (F g^–1^), energy density (E, mW h g^– 1^) and power density (P, mW g^–1^) of the supercapacitor device were calculated according to eqs 2, 3, and 4:^36,40^

$$C_{cell}=\frac{I\Delta t}{M\Delta V}$$ 2

$$E=\frac{C_{\mathrm{cell}}{\left(\Delta V\right)}^{2}}{2\times 3.6}$$ 3

$$P=\frac{E\times 3600}{\Delta t}$$ 4

where C_cell_ is the specific capacitance of the cell (F g^– 1^), M represents the total mass of the two composite films–based electrodes in the capacitor (g), ΔV is the potential window (V), and Δt is the discharge time (s).

## 3 Results and Discussion

### 3.1 Synthesis and Characterizations

#### 3.1.1 Synthesis and Characterizations of TPBT-COF@CNT and TPBT-COF@rGO Composite Materials

To demonstrate that the COFs and corresponding composites were successfully prepared, multiple characterization methods were used to study their physical and chemical properties. First, FTIR spectroscopy confirmed the successful synthesis and structural composition of the TPBT-COF@CNT composite materials (TPBT@CNT-30, TPBT@CNT-50, and TPBT@CNT-70) (Figure S1a). The characteristic absorption bands of f-CNT appear at 3400–3600 cm^–^^1^, attributed to the OH stretching vibrations,^41^ and at 1569 cm^–^^1^, corresponding to the stretching vibrations of the graphitic structure.^42^ For TPBT-COF, the spectrum exhibits distinctive absorption bands at 1660 cm^–^^1^ (C = N stretching), 1504 cm^–^^1^ (triazine core stretching), and 810 cm^–^^1^ (C = S–C stretching). The successful synthesis of TPBT-COF@CNT composites is evidenced by the presence of both CNT and COF characteristic peaks in their respective spectra. The composite materials show weak OH stretching absorption peaks at 3400–3600 cm^–^^1^, indicating some unreacted functional groups remaining on the CNT surface. The prominent peaks at 1660 cm^–^^1^ (C = N), 1504 cm^–^^1^ (triazine core), and 810 cm^–^^1^ (C = S–C) confirm the successful formation of the TPBT-COF structure within the composite. Additionally, the persistence of the graphitic structure stretching peak at 1569 cm^–^^1^ demonstrates the integration of f-CNT within the TPBT@CNT materials. Similarly, the FTIR spectra of rGO-based composites (TPBT@rGO-30, TPBT@rGO-40, and TPBT@rGO-50) were analyzed (Figure S1b). The reduced graphene oxide (rGO) spectrum shows characteristic absorption bands at 3400–3600 cm^–^^1^ (OH stretching), 1569 cm^–^^1^ (graphitic structure stretching),^42^ and 1077 cm^–^^1^ (C–O–C stretching).^43^ In the TPBT@rGO composite spectra, the presence of OH stretching absorption at 3400–3600 cm^–^^1^ indicates unreacted functional groups on the rGO surface. The successful formation of the TPBT-COF structure within these composites is confirmed by the appearance of characteristic peaks at 1665 cm^–^^1^ (C = N stretching), 1508 cm^–^^1^ (triazine core stretching), and 810 cm^–^^1^ (C = S–C stretching). Furthermore, the presence of graphitic structure stretching vibrations at 1561 cm^–^^1^ and C–O–C stretching at 1090 cm^–^^1^ confirms the incorporation of rGO within the composite materials.^43^ Notably, the TPBT@rGO composites show a slight shift in the C = N stretching band (1665 cm^–^^1^) compared to TPBT@CNT composites (1660 cm^–^^1^), suggesting different electronic environments or bonding arrangements at the interface between the COF and carbon substrates. The spectroscopic evidence collectively confirms the successful synthesis of both TPBT-COF@CNT and TPBT-COF@rGO composite materials with retained structural integrity of both components, indicating effective integration of the COF with the carbon-based materials. The f-CNT exhibited characteristic absorption bands at 3400–3600 cm^–^^1^ (OH stretching) and 1569 cm^–^^1^ (graphitic structure), while TPBT-COF showed distinctive signals at 1660 cm^–^^1^ (C = N), 1504 cm^–^^1^ (triazine core), 810 cm^–^^1^ (C = S–C) and OH stretching (3400–3600 cm^–^^1^). With increasing CNT and GO content in the composites, the characteristic peaks associated with the TPBT-COF structure progressively diminished in intensity. This systematic attenuation of the COF signals provides strong evidence for significant interactions between the COF and the carbon nanomaterials, suggesting effective integration of the two components within the composite system.

X-ray photoelectron spectroscopy (XPS) was employed to analyze the chemical composition and bonding states of TPBT@CNT-30, TPBT@CNT-50, TPBT@CNT-70, TPBT@rGO-30, TPBT@rGO-40, and TPBT@rGO-50 composites. Survey spectra and elemental compositions are presented in (Figure S2) and (Table S1), respectively. High-resolution C 1s spectra of TPBT@CNT composites (Figure S3a-c) revealed multiple chemical environments: C = C–S (284.3 eV), conjugated C–C/C = C from TPBT-COF (284.4 eV), C–C from CNT (285.5 eV), C = N–C (286.5 eV), and C = O from CNT (288.4 eV).^44^ The corresponding N 1s spectra (Figure S3d-f) exhibited two characteristic peaks at 398.0 and 399.2 eV, attributed to C = N and C–N bonds, respectively.^45^ O 1s deconvolution of TPBT@CNT composites (Figure S4a-c) identified four distinct chemical states: C = O from TPBT-COF (531.4 eV), O = C–N interfacial bonds (531.7 eV), C–OH from CNT (532.7 eV), and C–O from CNT (533.5 eV).^46^ Notably, the presence of O = C–N bonds, resulting from the reaction between f-CNT carboxyl groups and monomer amine groups, confirmed chemical grafting between f-CNT and TPBT-COF. The O = C–N peak intensity decreased with increasing carbon content, reflecting reduced TPBT-COF density. S 2p spectra (Figure S4d-f) showed characteristic doublets at 163.3 eV (S 2p3/2) and 164.4 eV (S 2p1/2) corresponding to C–S bonds in TPBT-COF.^47^ Analysis of TPBT@rGO composites revealed similar C 1s environments (Figure S5a-c): C = C–S (284.3 eV), C–C/C = C from TPBT-COF (284.4 eV), C–C from rGO (285.5 eV), C = N–C (286.5 eV), and C = O from rGO (288.4 eV).^48^ The N 1s spectra (Figure S5d-f) exhibited peaks at 398.0 eV (C = N) and 399.2 eV (C–N), characteristic of imine and triazine bonds in TPBT-COF.^45^ the O 1s spectra (Figure S6a-c) exhibited e peak at 531.4 eV corresponds to the C = O bond in TPBT-COF, while the peak at 531.7 eV indicates the O = C–N bonding formed between TPBT-COF and rGO. Peaks at 532.6, 532.8, and 533.5 eV are attributed to C–OH, O–C–O, and C–O bonds on the rGO surface, respectively.^46^ Notably, the presence of the O = C–N peak confirms the chemical bonding between the −COOH groups on rGO and the -NH_2_ groups of the monomer, demonstrating that the interaction between rGO and TPBT-COF occurs through chemical bonding rather than physical adsorption. As the carbon material content increases, the concentration of TPBT-COF per unit decreases, resulting in a corresponding reduction in the O = C–N peak intensity. the S 2p spectra (Figure S6d-f) shows the peaks at 163.3 and 164.4 eV corresponds to the C–S bonds of TPBT-COF, representing S 2p3/2 and S 2p1/2 signals, respectively.^47^

Thermogravimetric analysis under nitrogen atmosphere revealed enhanced thermal stability of the composite materials, with decomposition behavior intermediate between pure TPBT-COF and the carbon substrates (Figure S7). The thermal stability demonstrated a positive correlation with carbon material content in both TPBT@CNT and TPBT@rGO systems. Notably, despite the typical decomposition of surface functional groups around 200 °C, incorporation of carbon nanomaterials into the TPBT-COF significantly enhances the thermal stability of the resulting composites. For the TPBT@CNT series, thermal resilience progressively improves as CNT content increases from 30% to 70%, evidenced by diminished weight loss percentages at elevated temperatures. Similarly, the TPBT@rGO composites demonstrate enhanced thermal stability with increasing rGO content (30% to 50%) compared to pristine TPBT-COF.

X-ray diffraction analysis revealed characteristic diffraction peaks of TPBT-COF at 2θ angles of 4.64°, 8.08°, 9.26°, and 12.30°, corresponding to the (100), (110), (200), and (210) crystal planes, respectively.^39^ The angular relationships confirmed a hexagonal crystal system structure. A broad peak at 25.5° (001) was attributed to π-π stacking in the COF layered structure, with an interlayer spacing of d = 3.5 Å calculated using Bragg’s law. The XRD pattern of f-CNT (Figure 1a) exhibited a broad diffraction peak at 2θ = 25.84°, which overlapped with the TPBT-COF (001) reflection. Consequently, despite the decreased COF diffraction peak intensity with reduced TPBT-COF content in TPBT@CNT composites, the peak intensity in the 24–26° region showed a slight increase. The XRD pattern of rGO (Figure 1b) displayed a broad peak at 24°, reflecting the characteristic shift from 11.76° to 24–26° following reduction.^43^ This peak overlapped with the broad diffraction peak of COF in the same region. As rGO content increased, while the primary COF diffraction peaks decreased in intensity, the 24–26° region showed enhanced intensity.

**Figure 1:**
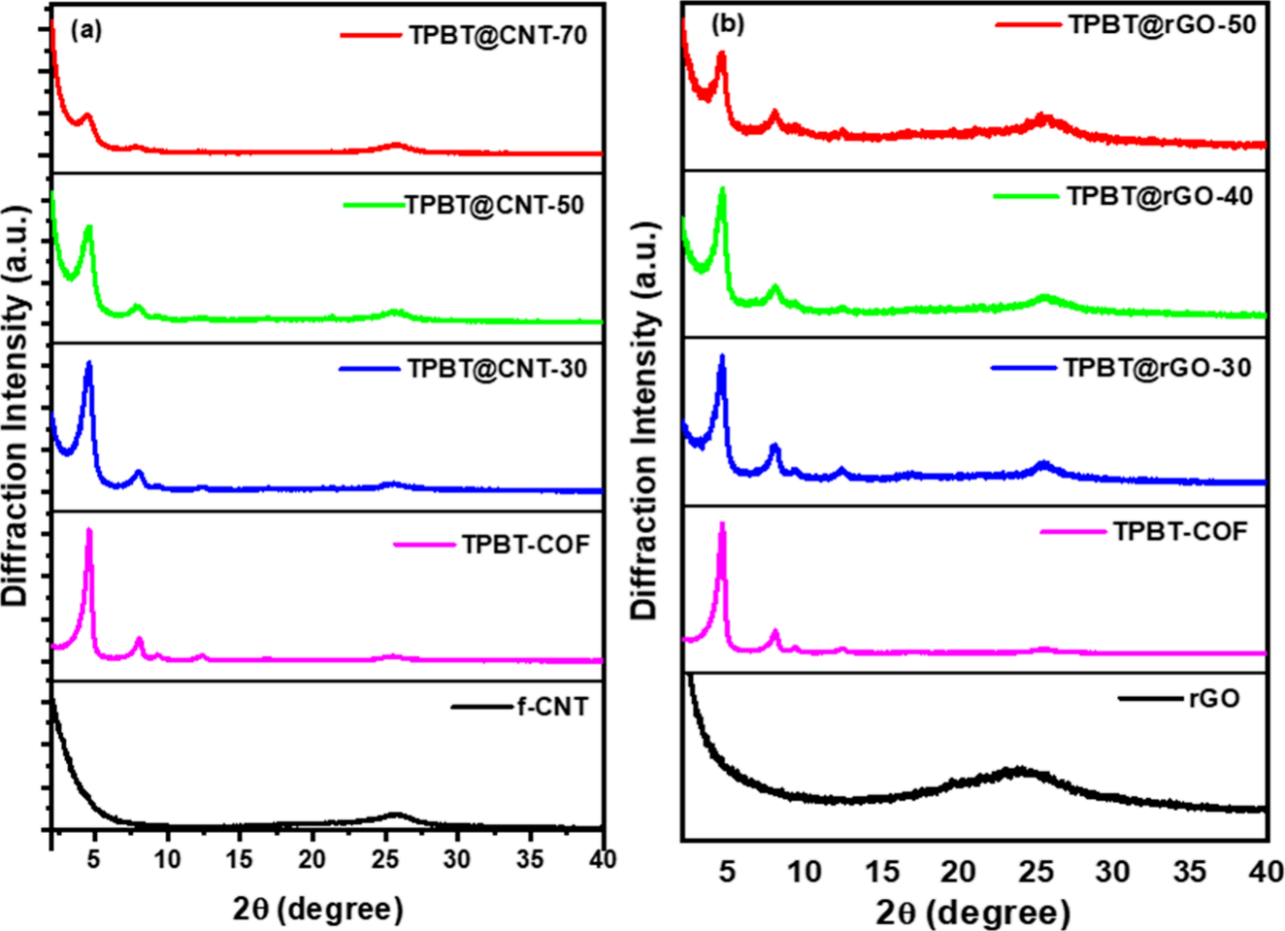
(a) XRD patterns of f-CNT, TPBT-COF, TPBT@CNT-30, TPBT@CNT-50, and TPBT@CNT-70; (b) XRD patterns of rGO, TPBT-COF, TPBT@rGO-30, TPBT@rGO-40, and TPBT@rGO-50.

SEM analysis (Figure 2) revealed distinct morphological features across the composite series. The precursor materials showed characteristic morphologies: sheet-like structure with wrinkled surfaces for GO, fibrous morphology for f-CNT, and needle-like structures with evident agglomeration for TPBT-COF (Figure S8). In TPBT@CNT composites, the presence of carboxyl functional groups on f-CNT surfaces effectively mitigated π-π stacking between COF layers, promoting secondary interactions with the f-CNT surface. The TPBT-COF layer thickness exhibited inverse correlation with f-CNT content, while maintaining uniform surface coverage. TPBT@rGO composites demonstrated similar morphological control, with oxygen-containing functional groups on GO surfaces facilitating uniform TPBT-COF distribution and preventing agglomeration. The incomplete surface coverage, attributed to GO’s high specific surface area and epoxy functional groups, maintained uniform distribution patterns across varying compositions.

**Figure 2:**
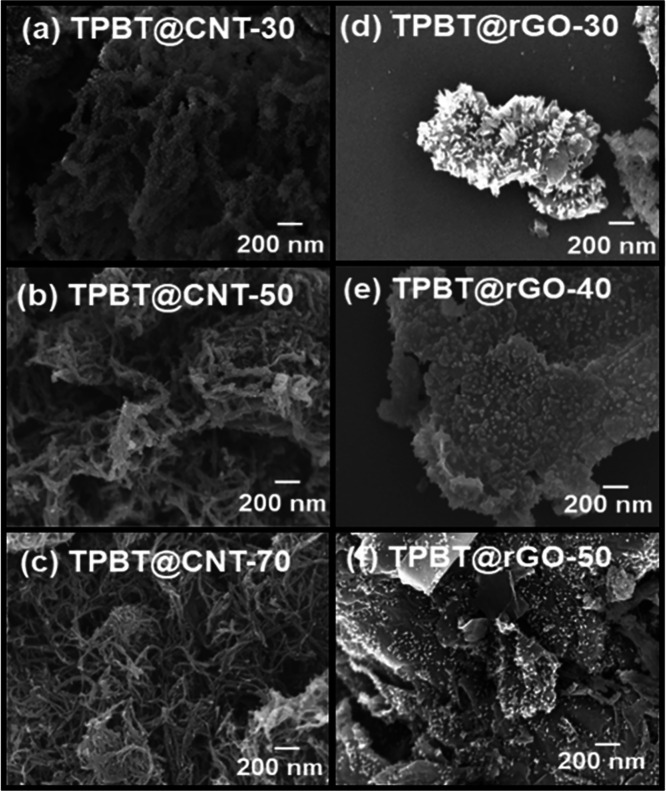
SEM images of (a) TPBT@CNT-30, (b) TPBT@CNT-50, (c) TPBT@CNT-70, (d) TPBT@rGO-30, (e) TPBT@rGO-40, and (f) TPBT@rGO-50.

TEM analysis of TPBT@CNT composites at various ratios (Figure 3 a-c) reveals that TPBT-COF (pure form shown in Figure S8c) uniformly encapsulates the exterior surface of f-CNT (pure CNT shown in Figure S8a), with the needle-like morphology of TPBT-COF exhibiting a shortening trend as f-CNT content increases, suggesting preferential physical stacking and reactivity with f-CNT rather than self-stacking; similarly, TEM micrographs of TPBT@rGO composites (Figure 3 d-f) demonstrate uniform growth of TPBT-COF on both rGO surfaces (pure rGO shown in Figure S8b), where increasing rGO content correlates with more prevalent vacant sites (semitransparent regions) on the rGO surface due to the relatively lower proportion of TPBT-COF available to interact with the functional groups and provide complete coverage, while the growth of TPBT-COF simultaneously facilitates exfoliation of the otherwise tightly stacked GO layers, resulting in nearly transparent rGO in regions lacking TPBT-COF growth.

**Figure 3:**
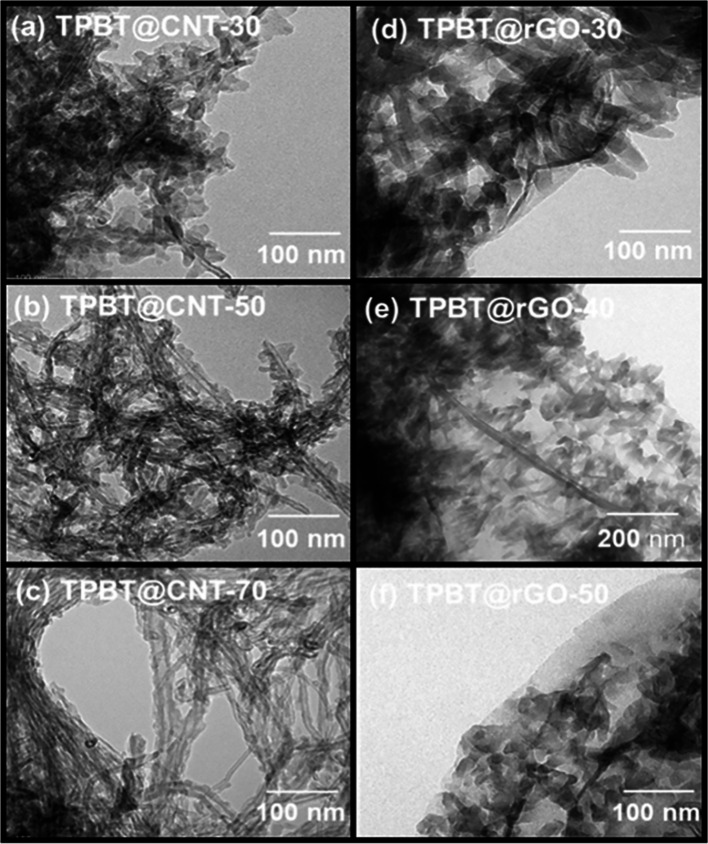
TEM images of (a) TPBT@CNT-30, (b) TPBT@CNT-50, (c) TPBT@CNT-70, (d) TPBT@rGO-30, (e) TPBT@rGO-40, and (f) TPBT@rGO-50.

The isothermal adsorption–desorption curves obtained through BET analysis for both composite systems are presented in Figure 4 (a,b). TPBT@CNT and TPBT@rGO composites exhibit rapidly increasing adsorption at low relative pressures, indicating the presence of micropores in these materials. These profiles represent typical Type I adsorption isotherms, characteristic of microporous materials with limited external surface area. As detailed in Table S2, the specific surface areas of both TPBT@CNT and TPBT@rGO composites demonstrate a decreasing trend with increasing carbon material content (CNT or rGO). This phenomenon can be attributed to the reduction in COF content per unit area, as the carbon-based materials contribute less to the overall surface area compared to the highly porous TPBT-COF structure. The pore size distributions derived using the Horvath–Kawazoe (H–K) model are illustrated in Figure 4 (c,d). These analyses reveal that the main pore diameters of TPBT@CNT and TPBT@rGO are approximately 0.7 and 1.15 nm, respectively, which are notably smaller than the 1.21 nm pore size observed in pure TPBT-COF. Additionally, the emergence of new pore populations is evident in the composite materials. The observed reduction in pore size can be explained by the structural organization at the interface between TPBT-COF and the carbon materials. The carbon substrates promote uniform and dense growth of TPBT-COF on their surfaces,^49^ creating a well-integrated composite structure. When TPBT-COF is grafted onto the carbon materials, the portion of the COF structure in direct contact with the carbon substrate becomes positionally constrained. This constraint induces distortion and misalignment in the π-π stacking arrangement of TPBT-COF, thereby reducing the main pore diameter. However, the TPBT-COF regions that are more distant from the carbon material interface retain greater structural flexibility and can undergo reversible redox-induced structural adjustments, resulting in pore characteristics more closely resembling those of pure TPBT-COF.

**Figure 4:**
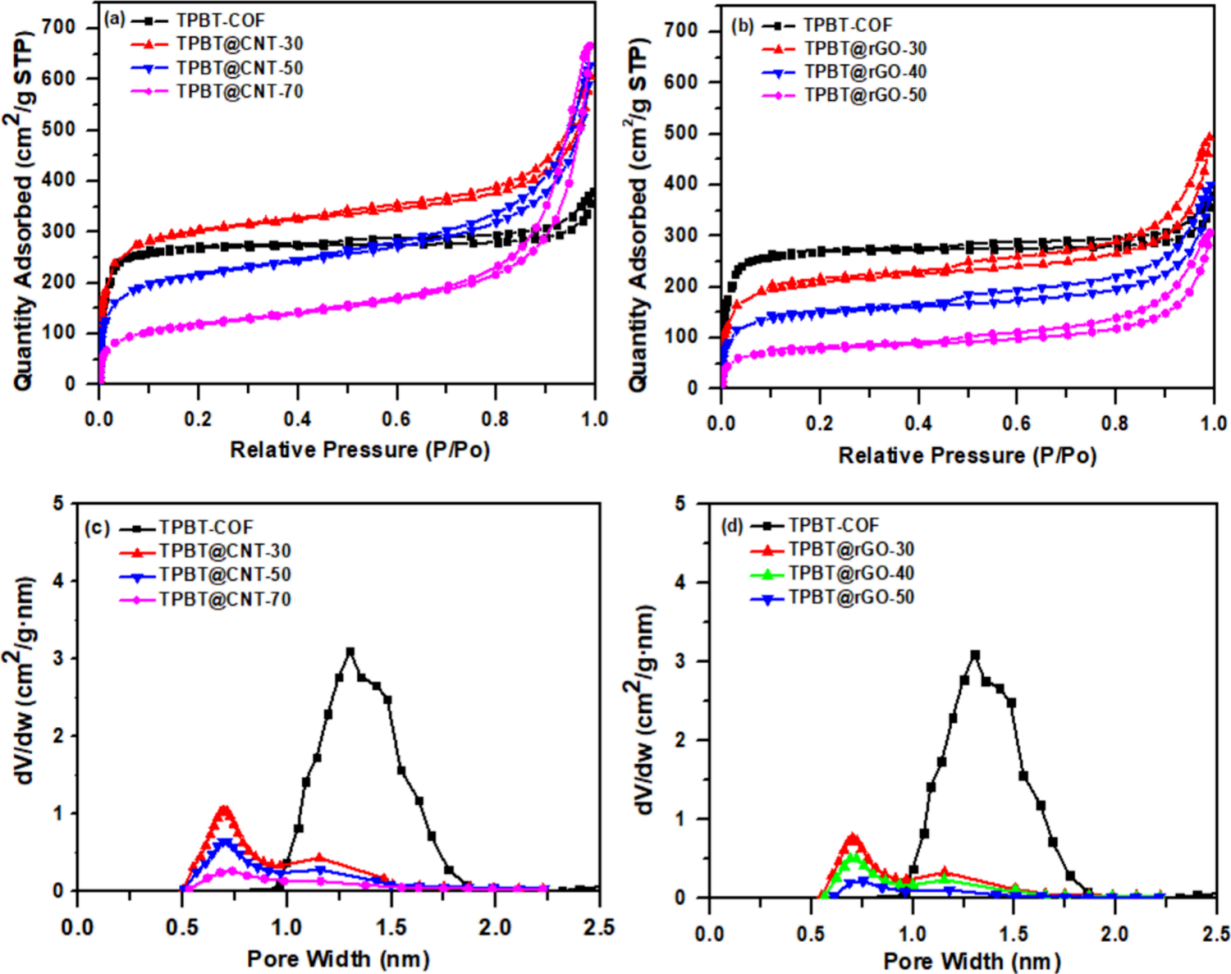
(a) Isothermal adsorption–desorption curves of TPBT@CNT and (b) TPBT@rGO composite materials; (c) Pore size distribution of TPBT@CNT and (d) TPBT@rGO composite materials.

#### 3.1.2 Characterizations of TPBT-COF@CNT/CNT/RC and TPBT-COF@rGO/rGO/RC Composite Films

SEM of the TPBT@CNT-50/CNT/RC composite reveals optimal microstructural organization, characterized by a pervasive CNT network architecture embedded within the cellulose framework (Figure S9). The synergistic interaction between the elongated CNT geometry and the fibrillar cellulose structure facilitates superior interfacial adhesion and promotes effective matrix integration (Figure S10a). This morphological compatibility results in homogeneous TPBT-COF deposition along the CNT periphery, establishing a well-ordered composite architecture. The cylindrical CNT geometry generates favorable void spaces throughout the cellulose network, simultaneously mitigating particle agglomeration and preserving essential ionic conductivity pathways. Conversely, the TPBT@rGO/rGO/RC composites demonstrate compromised microstructural integrity within the cellulose environment (Figure S9). Microscopic analysis identifies pronounced heterogeneity, manifesting as agglomerated rGO domains with irregular TPBT-COF spatial distribution. The inherent geometric incompatibility between the laminar rGO structure and the three-dimensional cellulose network promotes phase segregation over uniform blending (Figure S10b). This structural mismatch generates compositionally distinct microdomains, featuring densely aggregated rGO clusters interspersed with cellulose-dominated regions. Such morphological deficiencies severely compromise electrolyte infiltration efficiency and disrupt the establishment of continuous electron transport networks.

The XRD patterns of the TPBT-COF@CNT/CNT/RC and TPBT-COF@rGO/rGO/RC composite films are presented in Figure S11. Diffraction peaks corresponding to TPBT-COF can be observed in the range of 2θ = 4–12.5°, while those of RC appear in the range of 2θ = 12–22°. A strong peak of f-CNT and rGO was observed at 2θ = 25.84°, which overlapped with the TPBT-COF (001) reflection.

### 3.2 Electrochemical Performance of TPBT-COF@CNT/CNT/RC and TPBT-COF@rGO/rGO/RC Composite Film Electrodes for Supercapacitors

The CV curves, capacitance retention curves, and EIS spectra of CNT/RC (7:3, w/w) and rGO/RC (7:3, w/w) composite films are presented in Figure S12. The results indicate that the CNT/RC film-based electrode exhibits a higher capacitance value and better capacitance retention compared with the rGO/RC film-based electrode, while also showing a lower resistance. CNTs generally exhibit higher capacitance than rGO due to their superior electrical conductivity, open tubular structure, and efficient ion transport pathways. In contrast, the sheet-like morphology of rGO tends to restack, reducing the accessible surface area, while their lower conductivity and more tortuous ion diffusion paths further limit electrochemical performance. Consequently, CNT-based electrodes provide better charge storage compared with rGO.

The electrochemical properties of TPBT@CNT and TPBT@rGO composite films were systematically investigated to develop high-performance flexible electrodes for energy storage applications. Composite films with various weight ratios of TPBT@CNT/CNT/RC and TPBT@rGO/rGO/RC were fabricated and characterized using a comprehensive suite of electrochemical techniques. As shown in Figure S13, CV and GCD analyses were conducted on various composite formulations. The composite with a TPBT@CNT-50/CNT/RC ratio of 35/35/30 (w/w) exhibited maintained good shape retention even at higher scan rates, indicating excellent rate capability and efficient charge transfer kinetics, delivering a specific capacitance of 550.0 F/g at a current density of 1 A/g (Figure S14a). Electrochemical impedance spectroscopy (EIS) revealed significant insights into the interfacial properties of the composites. Figure S14b presents their corresponding Nyquist plots, characterized by a semicircle and a straight line in the high- and low-frequency regions, respectively. The radius of the semicircle represents the charge transfer resistance (R_ct_) and the slope of a straight line corresponds to the diffusion resistance of electrolyte ions. The intercept of the high-frequency semicircle with the *X*-axis is associated with the equivalent series resistance associated with the equivalent series resistance (R_S_).^47^ The TPBT@CNT-50/CNT/RC (70/0/30) formulation exhibits the largest semicircular arc, indicating substantial charge transfer resistance (Rct ≈ 25 Ω) and compromised electron transfer kinetics due to insufficient conductive pathways. The TPBT@CNT-50/CNT/RC (46.7/23.3/30) composition shows markedly reduced impedance with a smaller semicircle and improved low-frequency response, while the TPBT@CNT-50/CNT/RC (35/35/30) material demonstrates optimal impedance characteristics with an ideal balance between charge transfer resistance and ion diffusion properties, evidenced by a moderate semicircle and the most pronounced vertical line in the low-frequency region. In contrast, the TPBT@CNT-50/CNT/RC (0/70/30) sample exhibits low charge transfer resistance but limited capacitive behavior with a truncated response curve. These impedance profiles directly correspond to the electrochemical performance trends observed in CV and GCD measurements, confirming that the 35/35/30 composition achieves an optimal synergistic balance between electronic conductivity from pristine CNTs and pseudocapacitive contributions from TPBT-functionalized CNTs, resulting in superior supercapacitor performance through minimized electronic and ionic transport resistances.

#### 3.2.1 Electrochemical Performance of TPBT@CNT/CNT/RC and TPBT@rGO/rGO/RC Composite Film Electrodes

To further elucidate the electrochemical behavior of the TPBT@CNT/CNT/RC and TPBT@rGO/rGO/RC composite films, CV measurements were conducted in a three-electrode configuration within a potential window of −0.2 to 0.8 V in 1 M H_2_SO_4_ electrolyte at various scan rates (5–50 mV/s) (Figure 5). The curves exhibit quasi-rectangular shapes with visible redox peaks, indicating a combination of electric double-layer capacitance from carbon materials (CNT or rGO) and pseudocapacitance from the TPBT-COF. In all cases, increasing scan rates lead to expanded CV curve areas and slightly distorted shapes, reflecting diffusion limitations at higher rates. The TPBT@CNT demonstrate superior capacitive properties with larger enclosed areas in their CV curves, suggesting better electrical conductivity and electroactive surface area. EIS analysis (Figure S15) provided further evidence of the superior electrical properties of the TPBT@CNT-based composite. The TPBT@CNT/CNT/RC (35/35/30, w/w) exhibited significantly lower charge transfer resistance compared to its TPBT@rGO/rGO/RC (35/35/30, w/w) counterpart, confirming enhanced and improved ionic/electronic conductivity. The Rs and Rct values of the TPBT@CNT/CNT/RC composite films decreased with a reduction in the TPBT-COF deposition content on CNT. Specifically, the Rs values of TPBT@CNT-30/CNT/RC, TPBT@CNT-50/CNT/RC, and TPBT@CNT-70/CNT/RC were 10.1, 6.0, and 5.8 Ω, respectively, while the corresponding Rct values were 7.2, 6.6, and 2.7 Ω. In addition, the Rs values of TPBT@rGO-30/rGO/RC, TPBT@rGO-50/rGO/RC, and TPBT@rGO-70/rGO/RC were 80.1, 9.2, and 17.1 Ω, respectively, while the corresponding Rct values were 56.3, 65.1, and 35.5 Ω. The aggregation of TPBT-COF on CNT and rGO led to higher Rs and Rct values for the TPBT@CNT/CNT/RC and TPBT@rGO/rGO/RC composite films with increased TPBT-COF content.

**Figure 5:**
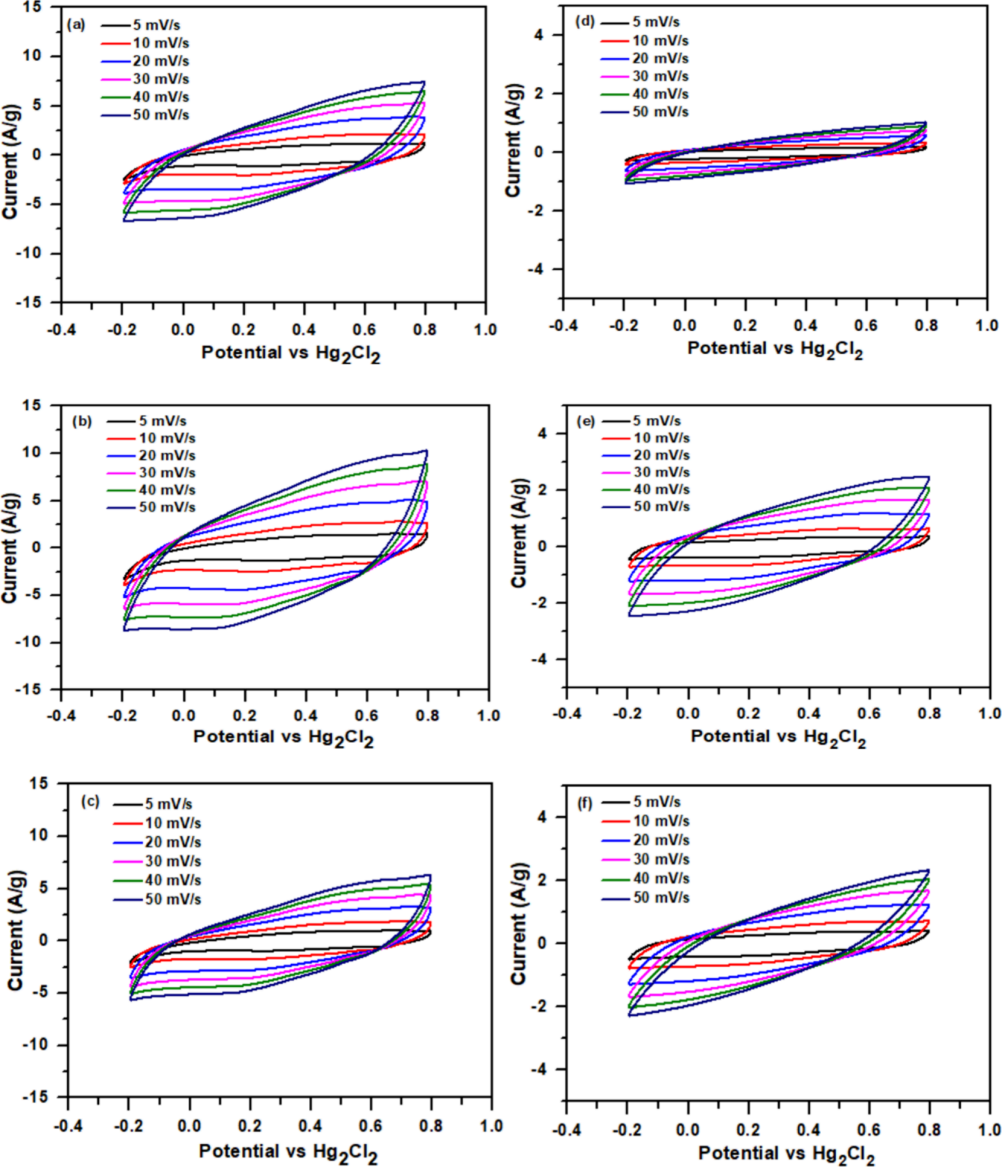
CV curves of composite films: (a) TPBT@CNT-30/CNT/RC (35/35/30, w/w), (b) TPBT@CNT-50/CNT/RC (35/35/30, w/w), (c) TPBT@CNT-70/CNT/RC (35/35/30, w/w), (d) TPBT@rGO-30/rGO/RC (35/35/30, w/w), (e) TPBT@rGO-40/rGO/RC (35/35/30, w/w), and (f) TPBT@rGO-50/rGO/RC (35/35/30, w/w).

GCD measurements conducted at current densities ranging from 0.5 to 5 A/g within a potential window of 0–0.8 V (Figure 6) demonstrated a direct correlation with the CV results. The TPBT@CNT/CNT/RC composites exhibited remarkable capacitive properties, achieving specific capacitances of 1182.36, 1288.26, and 1137.60 F/g at 0.5 A/g for TPBT@CNT-30, TPBT@CNT-50, and TPBT@CNT-70 compositions, respectively. These values substantially exceed those of the TPBT@rGO/rGO/RC composites, which delivered comparatively lower capacitances of 227.42, 335.50, and 398.75 F/g for TPBT@rGO-30, TPBT@rGO-40, and TPBT@rGO-50 compositions, respectively. Analysis of rate capability revealed that the TPBT@CNT-50/CNT/RC (35/35/30, w/w) achieved the highest specific capacitance of 1288.26 F/g at 0.5 A/g among all tested compositions, while TPBT@rGO-50/rGO/RC (35/35/30, w/w) exhibited the best performance among rGO-based composites with 398.75 F/g at 0.5 A/g. The superior performance of the TPBT@CNT-based composites can be attributed to several factors: (1) the one-dimensional tubular structure of CNTs facilitates more efficient electron transport compared to the two-dimensional sheet-like morphology of rGO; (2) CNTs create a more interconnected conductive network within the cellulose matrix; and (3) The dispersion characteristics of TPBT-COF within the composite matrices demonstrate a pronounced dependence on the carbonaceous substrate employed (Figure S16). For TPBT@CNT/CNT/RC systems, an inverse relationship exists between TPBT@CNT loading ratios and TPBT-COF aggregation phenomena, with higher ratios promoting homogeneous distribution across CNT surfaces. In contrast, TPBT@rGO/rGO/RC composites display persistent TPBT-COF clustering, originating from the intrinsic stacking behavior of graphene oxide layers. Apart from that, COF-based electrodes exhibit exceptionally high capacitance at low charge/discharge current densities, due to their highly porous framework and large specific surface area, which allow efficient ion diffusion and full utilization of electroactive sites. However, at higher current densities, the capacitance drops sharply, likely because the rapid charge/discharge process limits ion accessibility to the inner pores, reducing the effective utilization of the electrode material.

**Figure 6:**
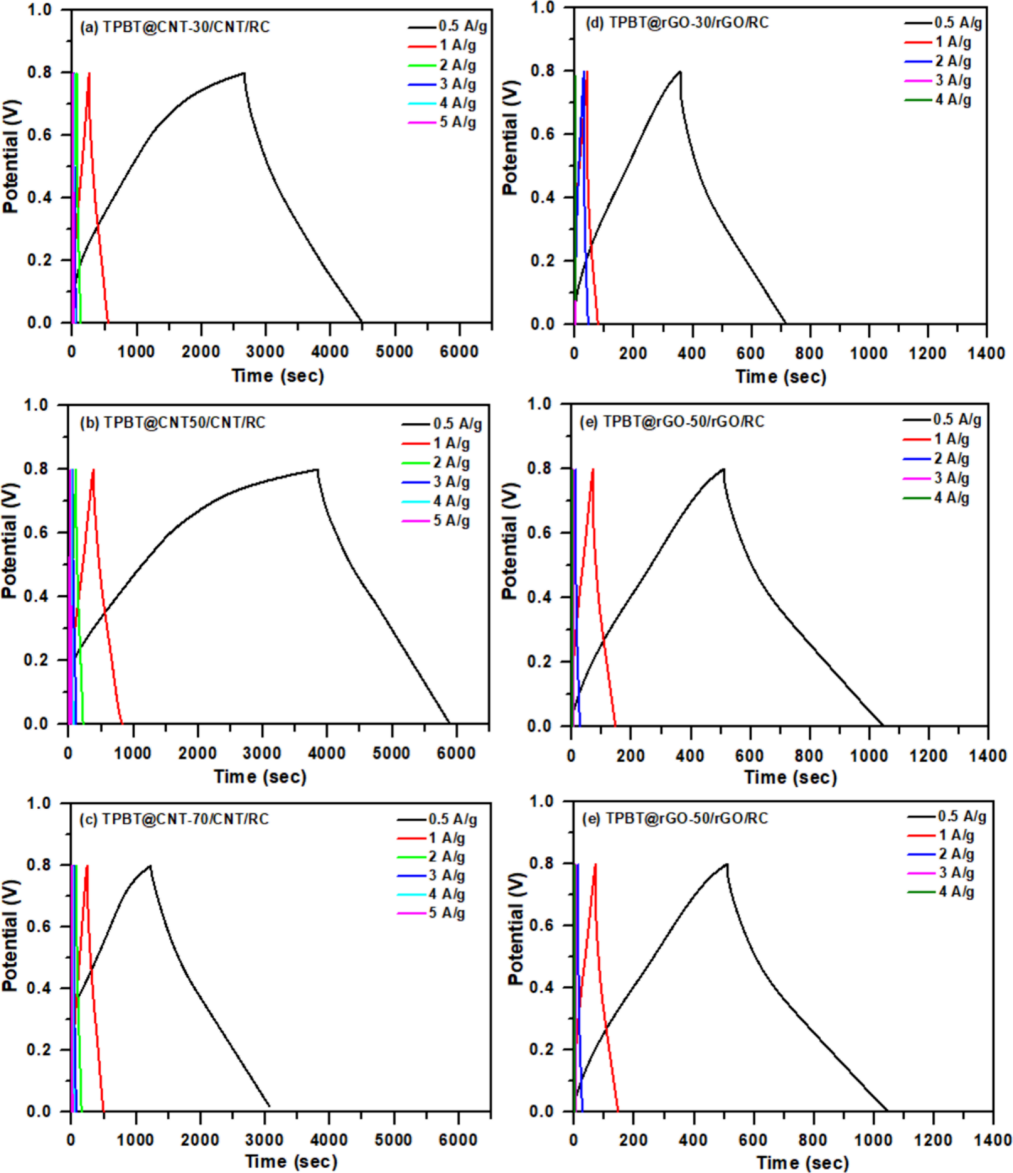
GCD curves of composite films: (a) TPBT@CNT-30/CNT/RC (35/35/30, w/w), (b) TPBT@CNT-50/CNT/RC (35/35/30, w/w), (c) TPBT@CNT-70/CNT/RC (35/35/30, w/w), (d) TPBT@rGO-30/rGO/RC (35/35/30, w/w), (e) TPBT@rGO-40/rGO/RC (35/35/30, w/w), and (f) TPBT@rGO-50/rGO/RC (35/35/30, w/w).

Based on the GCD curves (Figure 6), the Coulombic efficiency (CE) can be directly calculated from the ratio of discharge time to charge time. CE reflects the reversibility of the electrochemical process, with values near 100% signifying efficient charge recovery and minimal energy loss. Compared to the TPBT@CNT-70/CNT/RC electrode, the lower CE values of the TPBT@CNT-30/CNT/RC and TPBT@CNT-50/CNT/RC electrodes can be attributed to the aggregation and stacking of TPBT-COF on the CNTs, which hinder ion diffusion during charging. Longer charge times thus result in lower CE values, particularly at lower charge current densities (0.5 A/g). In contrast, the TPBT@rGO/rGO/RC composite films exhibit a high CE close to 100%, indicating that most of the charges stored during charging can be efficiently recovered during discharging. Nevertheless, these electrodes show relatively low capacitance, suggesting that the dominant contribution to the capacitance arises from the electric double-layer capacitance of the rGO rather than the TPBT-COF.

Long-term cycling stability is a critical parameter for practical energy storage applications. As shown in Figure 7(a), the capacitance decreased with increasing charge current density. When the current density exceeded 4 A g^–^^1^, the TPBT@rGO-50/rGO/RC electrode exhibited signs of structural degradation or even potential damage. In contrast, the TPBT@CNT-50/CNT/RC electrode was able to tolerate higher charge current densities. Therefore, the long-term cycling stability of the TPBT@CNT-50/CNT/RC and TPBT@rGO-50/rGO/RC electrodes were measured at current density of 10 and 4 A g^–^^1^, respectively. The TPBT@CNT-50/CNT/RC (35/35/30, w/w) composite film demonstrated exceptional electrochemical durability, retaining 85.87% of its initial capacitance after 10,000 charge–discharge cycles at a high current density of 10 A/g. In contrast, the TPBT@rGO-50/rGO/RC (35/35/30, w/w) composite film could only withstand current densities up to 4 A/g and retained 81.82% of its initial capacitance after 10,000 cycles at this lower current density (Figure 7b). The superior cycling stability of the TPBT@CNT-based composite can be attributed to its robust structural integrity during repeated charge–discharge processes. The one-dimensional tubular structure of CNTs provides enhanced mechanical support to the TPBT-COF framework, preventing structural collapse during prolonged cycling. Additionally, the stronger interaction between TPBT-COF and CNTs minimizes active material detachment from the electrode during cycling, thus preserving electrochemical performance over extended periods. Nevertheless, compared with the TPBT@CNT-50/CNT/RC (35/35/30, w/w) and TPBT@rGO-50/rGO/RC (35/35/30, w/w) electrodes before cycling (Figure 2 and Figure S9), the SEM images indicated that the amount of TPBT-COF deposited on CNT and rGO decreased after the charge–discharge cycling test (Figure S16), with a more pronounced reduction observed for the TPBT@rGO-50/rGO/RC sample.

**Figure 7:**
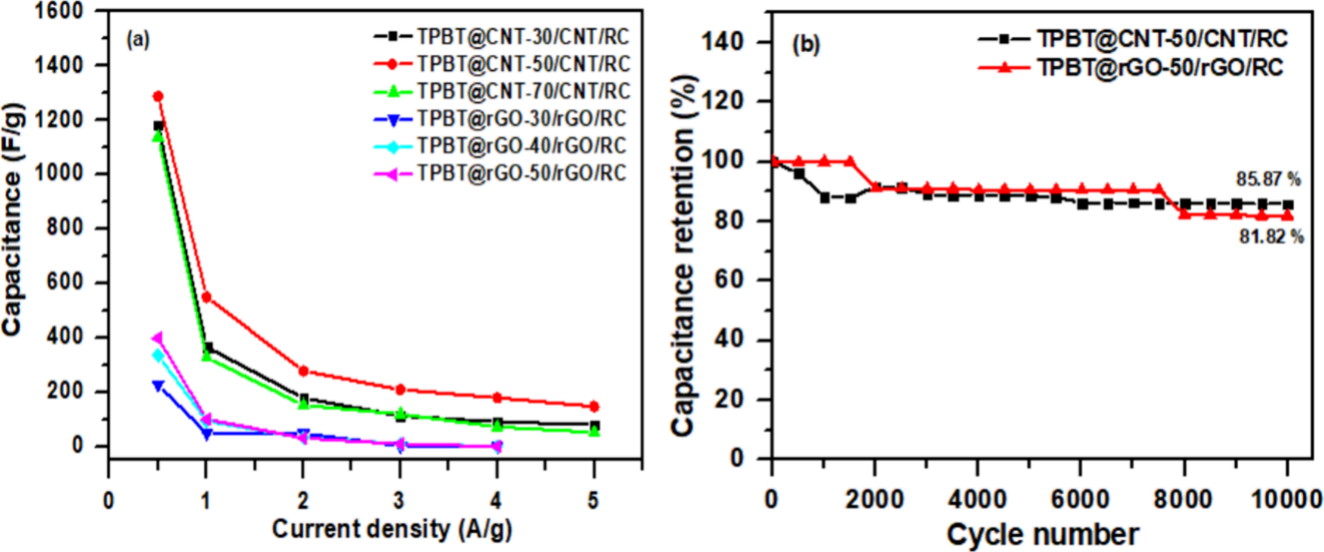
(a) Capacitance versus current density relationship of TPBT@CNT/CNT/RC (35/35/30, w/w) and TPBT@rGO/rGO/RC (35/35/30, w/w) composite films. (b) Cycling stability test of TPBT@CNT-50/CNT/RC (35/35/30, w/w) and TPBT@rGO-50/rGO/RC (35/35/30, w/w) composite films.

**Figure 8:**
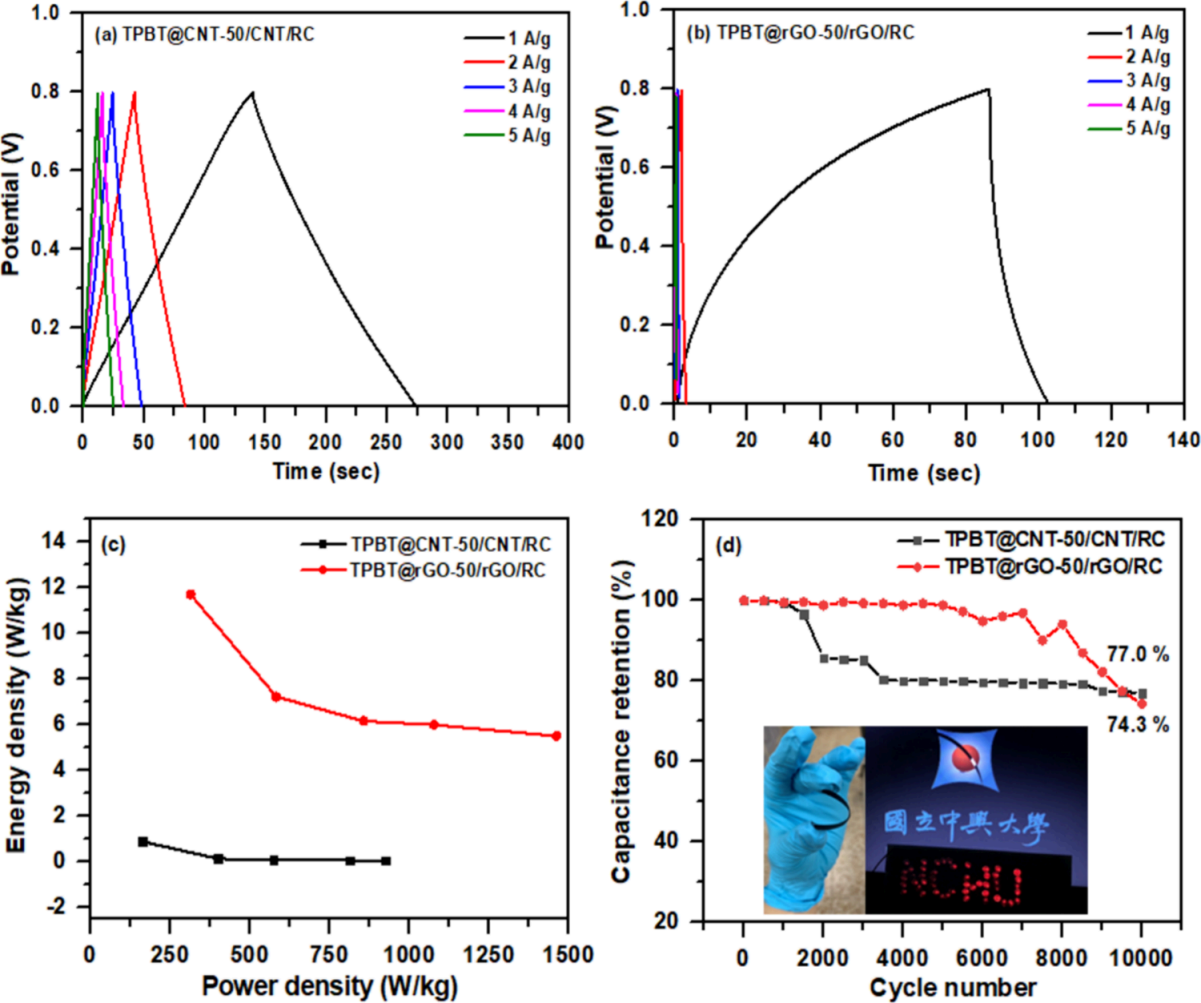
(a) GCD curves of symmetric devices fabricated with TPBT@CNT-50/CNT/RC (35/35/30, w/w) films, (b) GCD curves of symmetric devices fabricated with TPBT@rGO-50/rGO/RC (35/35/30, w/w) films, (c) energy density versus power density relationship of symmetric devices made with TPBT@CNT-50/CNT/RC (35/35/30, w/w) and TPBT@rGO-50/rGO/RC (35/35/30, w/w), (d) Cycle life of symmetric devices fabricated with TPBT@CNT-50/CNT/RC (35/35/30, w/w) and TPBT@rGO-50/rGO/RC (35/35/30, w/w) (the inset shows a digital photograph of the LED powered by the SSC, and flexibility of film).

#### 3.2.2 Electrochemical Performance of Symmetric Supercapacitors

To evaluate the practical application potential of the optimized composite films, symmetric supercapacitor devices were fabricated using TPBT@CNT-50/CNT/RC (35/35/30, w/w) and TPBT@rGO-50/rGO/RC (35/35/30, w/w) composite films as both positive and negative electrodes. The CV curves of the TPBT@CNT-based symmetric device (Figure S17a) exhibited a more rectangular shape compared to the three-electrode configuration, this rectangular shape is maintained even at higher scan rates, demonstrating rapid ion transport and efficient charge accumulation at the electrode–electrolyte interface. Additionally, the increased current response with rising scan rates without significant distortion in the CV curve shape suggests low internal resistance and fast electron transfer kinetics, which can be attributed to the synergistic effect between the TPBT-COF structure and the conductive CNT network established during the in situ polymerization process. In contrast, the TPBT@rGO-based symmetric device (Figure S17b) showed diminished capacitance compared to its three-electrode counterpart, which can be attributed to increased ionic diffusion resistance within the electrode structure. EIS analysis (Figure S17c) further confirmed the superior electrical properties of the TPBT@CNT-based symmetric device, exhibiting lower internal resistance and more efficient charge transfer kinetics compared to the TPBT@rGO-based device. The stacking of the sheet-like rGO reduces the accessible surface area, while its lower conductivity and more tortuous ion diffusion pathways further hinder charge transport within the composite film. As a result, the TPBT@rGO-based symmetric device exhibits higher internal resistance. The GCD measurements of symmetric devices revealed significant performance disparities between CNT and rGO-based composites (Figure 8a,b). The TPBT@CNT-50/CNT/RC (35/35/30, w/w) symmetric device delivered an impressive specific capacitance of 84.32 F/g at 1 A/g, substantially outperforming the TPBT@rGO-50/rGO/RC (35/35/30, w/w) symmetric device, which achieved only 10.07 F/g under identical conditions. Nevertheless, the capacitance values of the symmetric devices are lower than those of the TPBT@CNT/CNT/RC (35/35/30, w/w) and TPBT@rGO/rGO/RC (35/35/30, w/w) composite film electrodes (Figure 7a). This reduction is expected, since in a symmetric two-electrode configuration the two identical electrodes are connected in series, resulting in an overall device capacitance that is inherently half of the single-electrode capacitance measured in a three-electrode system.^50^ Furthermore, in practical two-electrode devices, additional ionic transport limitations can arise from the separator and the finite thickness of the electrolyte layer, which hinder ion diffusion between the electrodes. These effects increase internal resistance and reduce the utilization of the active material compared with three-electrode measurements. Analysis of energy and power density relationships (Figure 8c) demonstrated that the TPBT@CNT-50/CNT/RC device achieved maximum energy and power densities of 11.71 Wh/kg and 312.5 W/kg, respectively. These values significantly exceed those of the TPBT@rGO-50/rGO/RC device, which delivered maximum energy and power densities of 0.90 Wh/kg and 160 W/kg, respectively. The superior energy storage performance of the TPBT@CNT-based device can be attributed to its enhanced electrical conductivity, reduced charge transfer resistance, and more efficient electrode–electrolyte interface. The specific capacitance, energy density, and power density of the TPBT@CNT-50/CNT/RC electrode-based supercapacitor were found to be comparable to, or even superior to, those of recently reported triazine-COF/CNT and triazine-COF/GO supercapacitors (Table S3).^17,33,35,36,51^

The long-term electrochemical stability of the TPBT@CNT-50/CNT/RC- and TPBT@rGO-50/rGO/RC-based symmetric supercapacitors was evaluated over 10,000 charge–discharge cycles at current densities of 10 and 4 A g^–^^1^, respectively (Figure 8d). The TPBT@CNT-50/CNT/RC (35/35/30, w/w) symmetric device demonstrated superior cycling durability, retaining 77.0% of its initial capacitance after 10,000 cycles. In comparison, the TPBT@rGO-50/rGO/Cellulose (35/35/30, w/w) device retained 74.3% of its initial capacitance under the same testing conditions. A drop in capacitance retention was observed after 1,500–2,000 charge–discharge cycles for the TPBT@rGO-50/rGO/RC-based device. This degradation may be attributed to insufficient adhesion between the TPBT-COF and the rGO, where repeated cycling can cause interfacial delamination and consequently reduce the amount of effectively active material. The SEM image of the TPBT@rGO-50/rGO/RC film revealed that the amount of TPBT-COF deposited on rGO decreased after the charge–discharge cycling test (Figure 2f and Figure S16b). While both devices exhibited slightly lower stability compared to their respective electrodes in three-electrode configurations, the TPBT@CNT-based symmetric device maintained higher capacitance retention, further confirming its enhanced electrochemical stability for practical energy storage applications. The exceptional cycling stability of the TPBT@CNT-based device can be attributed to the synergistic integration of TPBT-COF with CNT, resulting in a robust composite structure capable of withstanding repeated charge–discharge cycles without significant degradation. What’s more, four symmetric device of TPBT@CNT-50/CNT/RC (35/35/30, w/w) connected in series can successfully light up a “NCHU” panel consisting of 49 LEDs.

#### 3.2.3 Structure–Property Relationships and Mechanistic Insights

The remarkable electrochemical performance disparities between TPBT@CNT and TPBT@rGO composite films can be explained by examining their fundamental structure–property relationships. The one-dimensional tubular structure of CNTs facilitates more efficient electron transport pathways compared to the two-dimensional sheet-like morphology of rGO. Additionally, CNTs create a more interconnected conductive network within the cellulose matrix, enhancing overall electrical conductivity and charge transfer kinetics. The superior performance of the TPBT@CNT-50/CNT/Cellulose (35/35/30, w/w) composite can be attributed to an optimal balance between active material (TPBT-COF) content and conductive additive (CNT) proportion. This optimized composition enables effective utilization of the high theoretical capacitance of TPBT-COF while maintaining efficient electron transport pathways through the CNT network. The synergistic integration of TPBT-COF with CNT in a cellulose matrix represents a significant advancement in the development of high-performance flexible electrodes for next-generation energy storage systems. The exceptional specific capacitance (1288.26 F/g at 0.5 A/g), excellent rate capability, and superior cycling stability (85.87% retention after 10,000 cycles) of the TPBT@CNT-50/CNT/RC (35/35/30, w/w) composite electrode demonstrate its potential for practical supercapacitor applications.

## 4 Conclusion

In conclusion, we have demonstrated successful synthesis of TPBT-COF grafted onto CNT and rGO substrates, effectively addressing COF aggregation challenges. The TPBT@CNT-50 composite exhibited exceptional thermal stability with a decomposition temperature of 577.43 °C, while microscopic analyses confirmed uniform TPBT-COF growth on carbon material surfaces. The grafting mechanism, verified through XPS analysis, induced structural modifications that resulted in distinctive pore size distributions, as evidenced by BET measurements. The electrochemical performance evaluation revealed that TPBT@CNT-50/CNT/Cellulose (35/35/30, w/w) composite electrodes achieved remarkable capacitive performance (1288.26 F/g at 0.5 A/g) with excellent cycling stability (85.87% retention after 10,000 cycles at 10 A/g). When assembled into symmetric supercapacitor devices, the CNT-based composite demonstrated superior energy and power densities (11.71 Wh/kg and 312.5 W/kg, respectively) compared to its rGO-based counterpart (0.90 Wh/kg and 160 W/kg). This enhanced performance can be attributed to the optimal TPBT-COF/CNT ratio, which maximizes interfacial contact while preventing aggregation, combined with superior dispersion and interaction with the cellulose matrix. These findings suggest that TPBT@CNT-based composites represent a promising direction for developing high-performance flexible energy storage devices.

## Supplementary Material



## Notes

FTIR spectra, XPS survey spectra, and TGA thermograms of the TPBT-COF@CNT and TPBT-COF@rGO composite films; TEM images of f-CNT, rGO, and TPBT-COF; Specific surface areas and pore size of the TPBT@CNT and TPBT@rGO composites; CV, GCD curves, and SEM images of the TPBT@CNT/CNT/RC and TPBT@rGO/rGO/RC electrodes; Capacitance versus current density and EIS diagrams of TPBT@CNT-50/CNT/RC electrodes. (PDF)
