# Supplementary material for: π‑Conjugated Triazine–Benzotrithiophene COF Networks Integrated with Carbon Nanotubes and Reduced Graphene Oxide in Cellulose Films for High-Performance Supercapacitors
Source: ACS Polym Au. 2025 Oct 20;5(6):930–43. doi: 10.1021/acspolymersau.5c00111 (PMC12874156; doi:10.1021/acspolymersau.5c00111)
Supplement: Supplementary file 1 [file lg5c00111_si_001.pdf]

# Supporting Information

# π-Conjugated Triazine–Benzotrithiophene COF Networks Integrated with Carbon Nanotubes and Reduced Graphene Oxide in Cellulose Films for High-Performance Supercapacitors

Yi-Yun Chen[1§], Mahmoud Younis[1,2§], Pei-Cih Hu[1], Peng-Yao Chen[1], Cheng-Yeh Hsin[1], Hongta Yang[1], Bo-Tau Liu[3], and Rong-Ho Lee*[1,4]

1. Department of Chemical Engineering, National Chung Hsing University, Taichung, 402, Taiwan
2. Chemistry Department, Faculty of Science, New Valley University, El-Kharja, 72511, Egypt.
3. Department of Chemical and Materials Engineering, National Yunlin University of Science and Technology, Yunlin 64002, Taiwan.
4. Department of Chemical Engineering and Materials Science, Yuan Ze University, Taoyuan City 320, Taiwan.

*To whom correspondence should be addressed.

§ These authors contributed equally to this work

Rong-Ho Lee—e-mail: rhl@nchu.edu.tw; tel.: +886-4-22854308; fax: +886-4-22854734.

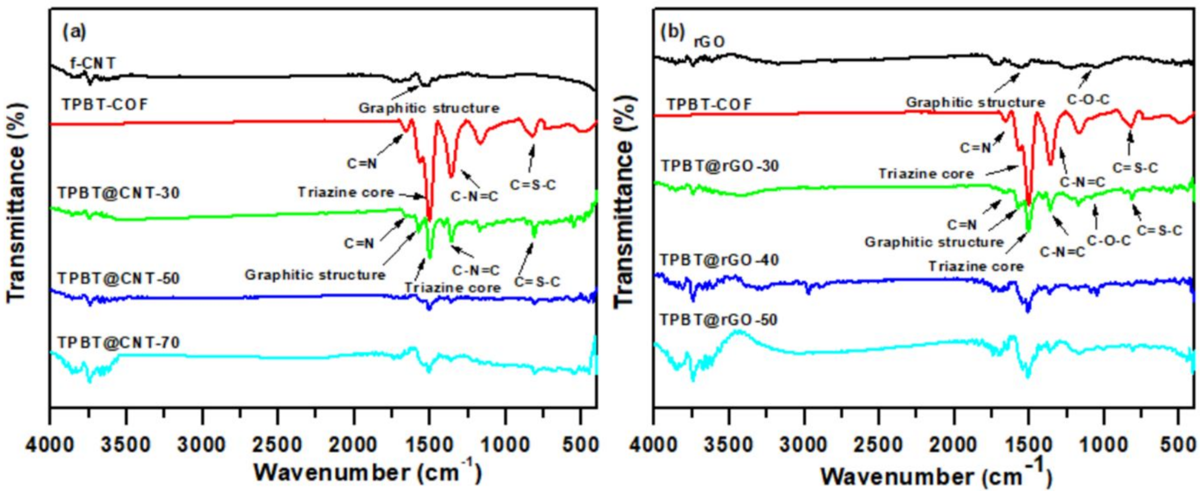


Figure S1. FTIR spectra of the (a) TPBT-COF@CNT and (b) TPBT-COF@rGO composite materials.

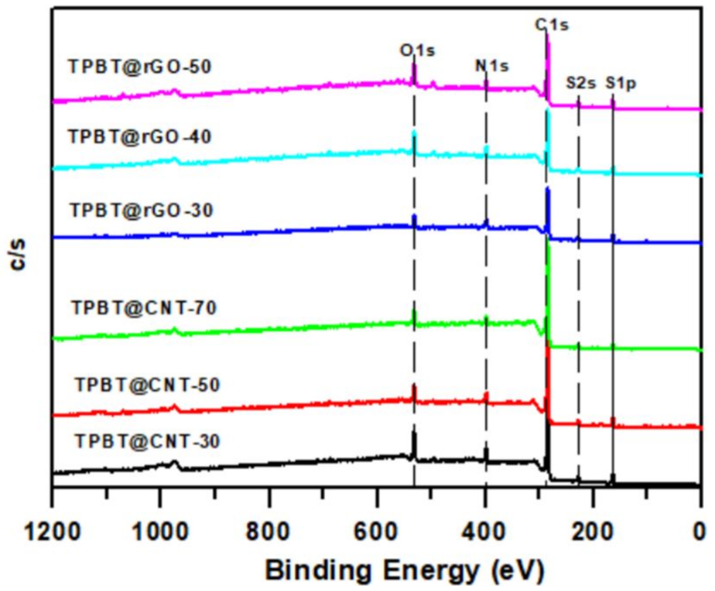


Figure S2. XPS survey spectra of TPBT@CNT and TPBT@rGO composite materials.

Table S1 Elemental composition of TPBT@CNT and TPBT@rGO composites at various ratios.

| | C 1s | N 1s | O 1s | S 2p |
|---|---|---|---|---|
| TPBT@CNT-30 | 75.4 % | 8.5 % | 12.7 % | 3.4 % |
| TPBT@CNT-50 | 82.8 % | 6.3 % | 7.9 % | 3.0 % |
| TPBT@CNT-70 | 87.3 % | 4.8 % | 6.2 % | 1.8 % |
| TPBT@rGO-30 | 78.7 % | 7.9 % | 9.4 % | 3.9 % |
| TPBT@rGO-40 | 75.2 % | 8.4 % | 13.0 % | 3.4 % |
| TPBT@rGO-50 | 76.5 % | 6.2 % | 14.2 % | 3.0 % |

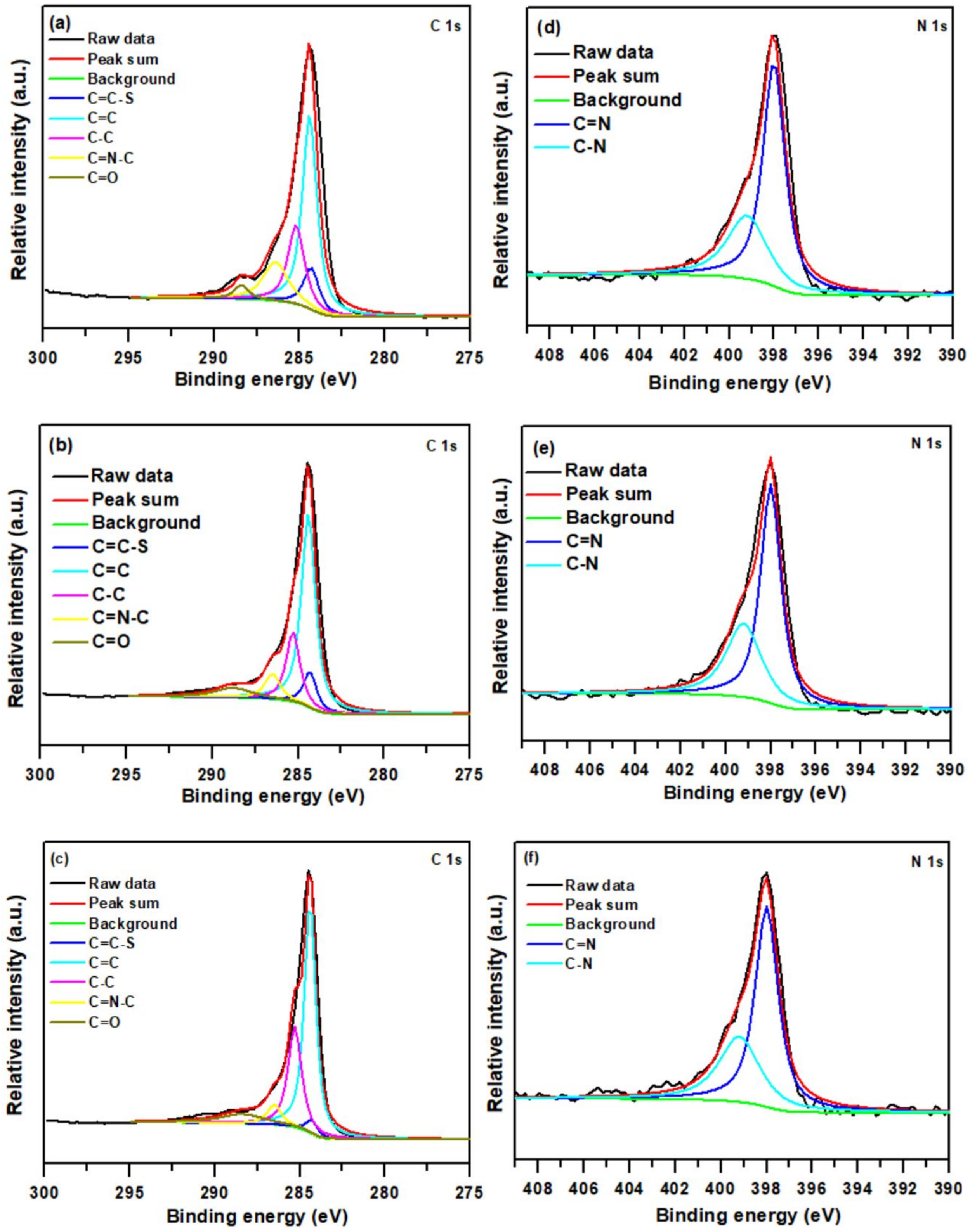


Figure S3. XPS spectra of C1s [(a) TPBT@CNT-30, (b) TPBT@CNT-50, and (c) TPBT@CNT-70] and N1s [(d) TPBT@CNT-30, (e) TPBT@CNT-50, and (f) TPBT@CNT-70].

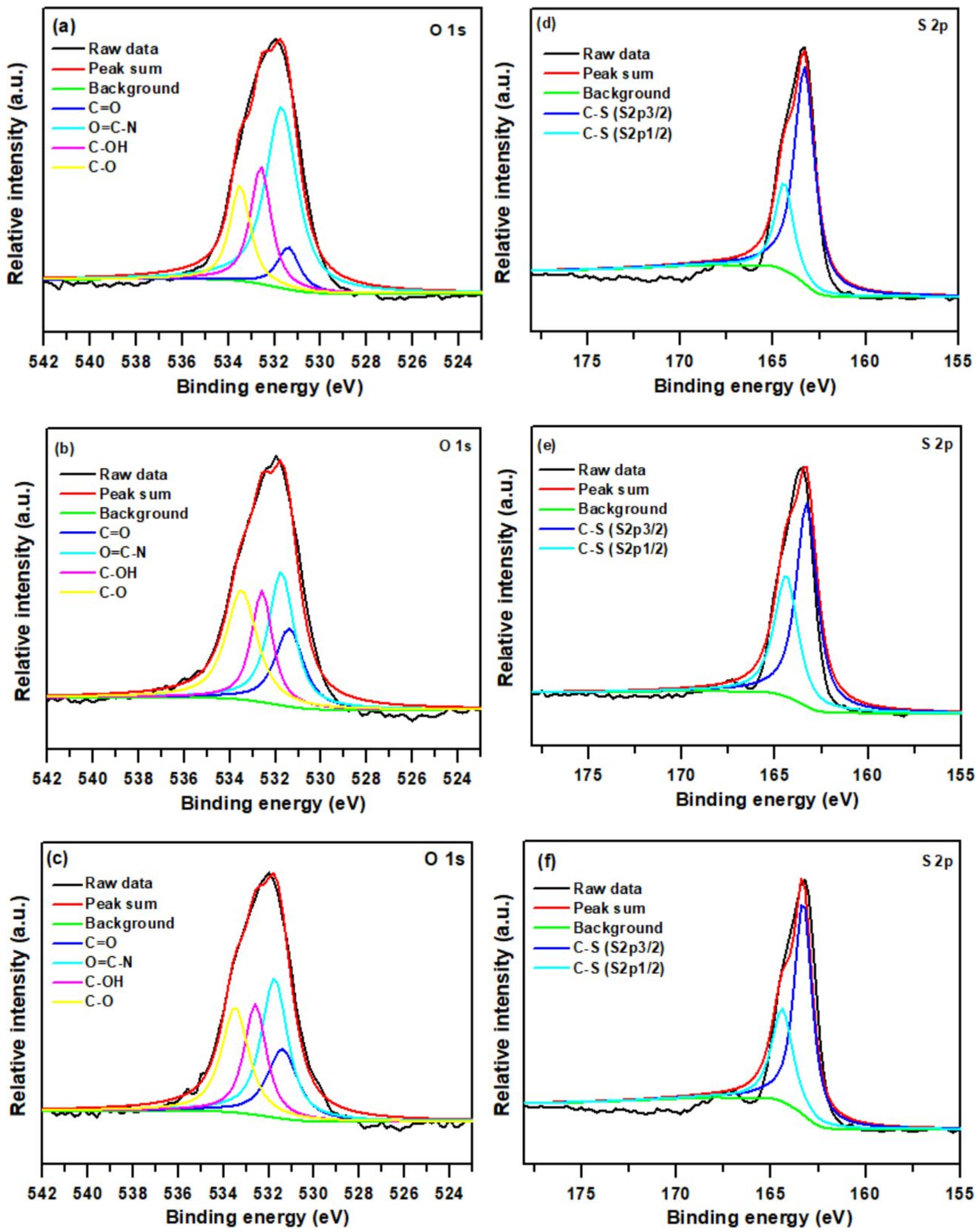


Figure S4. XPS spectra of O1s [(a) TPBT@CNT-30, (b) TPBT@CNT-50, and (c) TPBT@CNT-70] and S2p [(d) TPBT@CNT-30, (e) TPBT@CNT-50, and (f) TPBT@CNT-70].

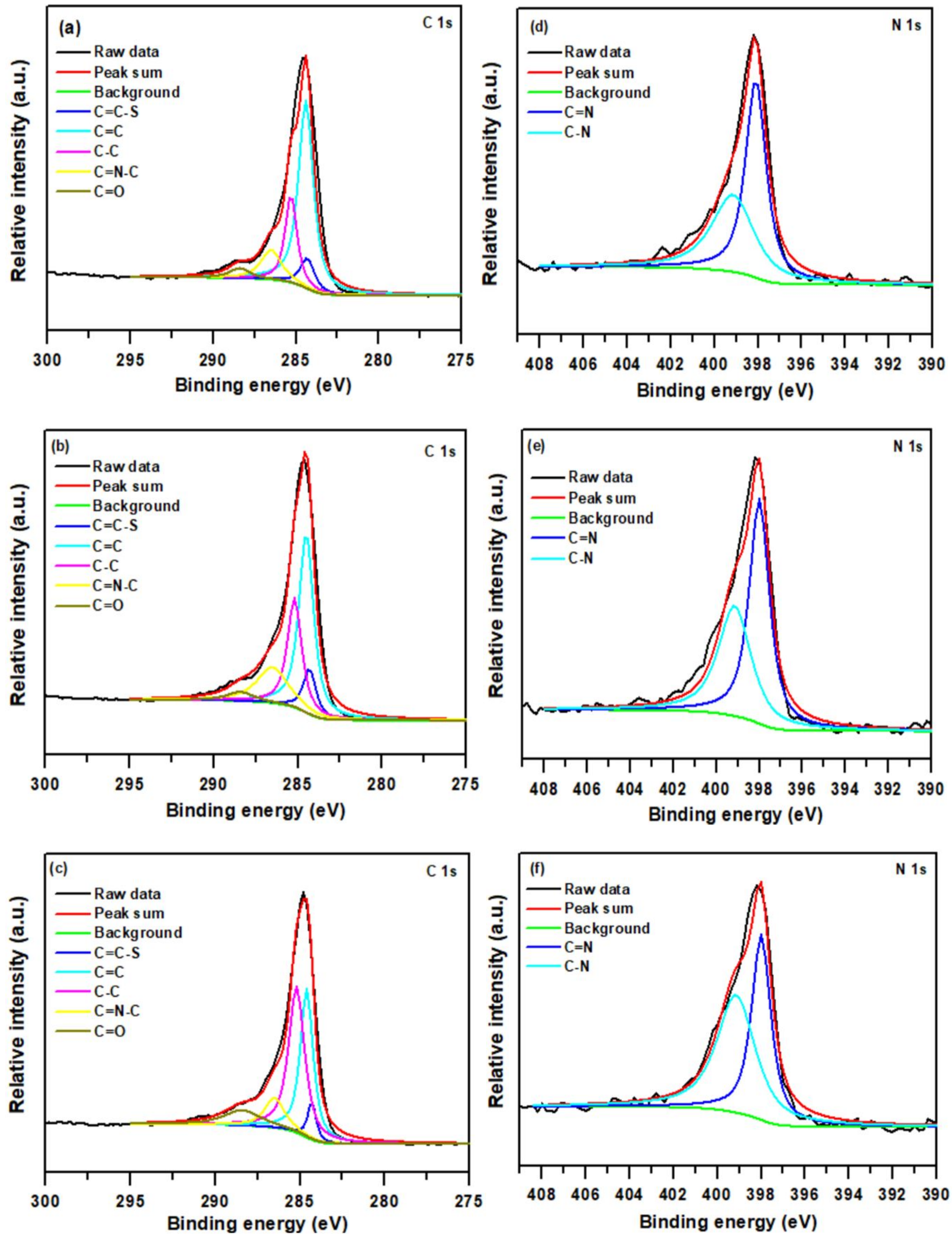


Figure S5. XPS spectra of C1s [(a) TPBT@rGO-30, (b) TPBT@rGO-40, and (c) TPBT@rGO-50] and N1s [(d) TPBT@rGO-30, (e) TPBT@rGO-40, and (f) TPBT@rGO-50].

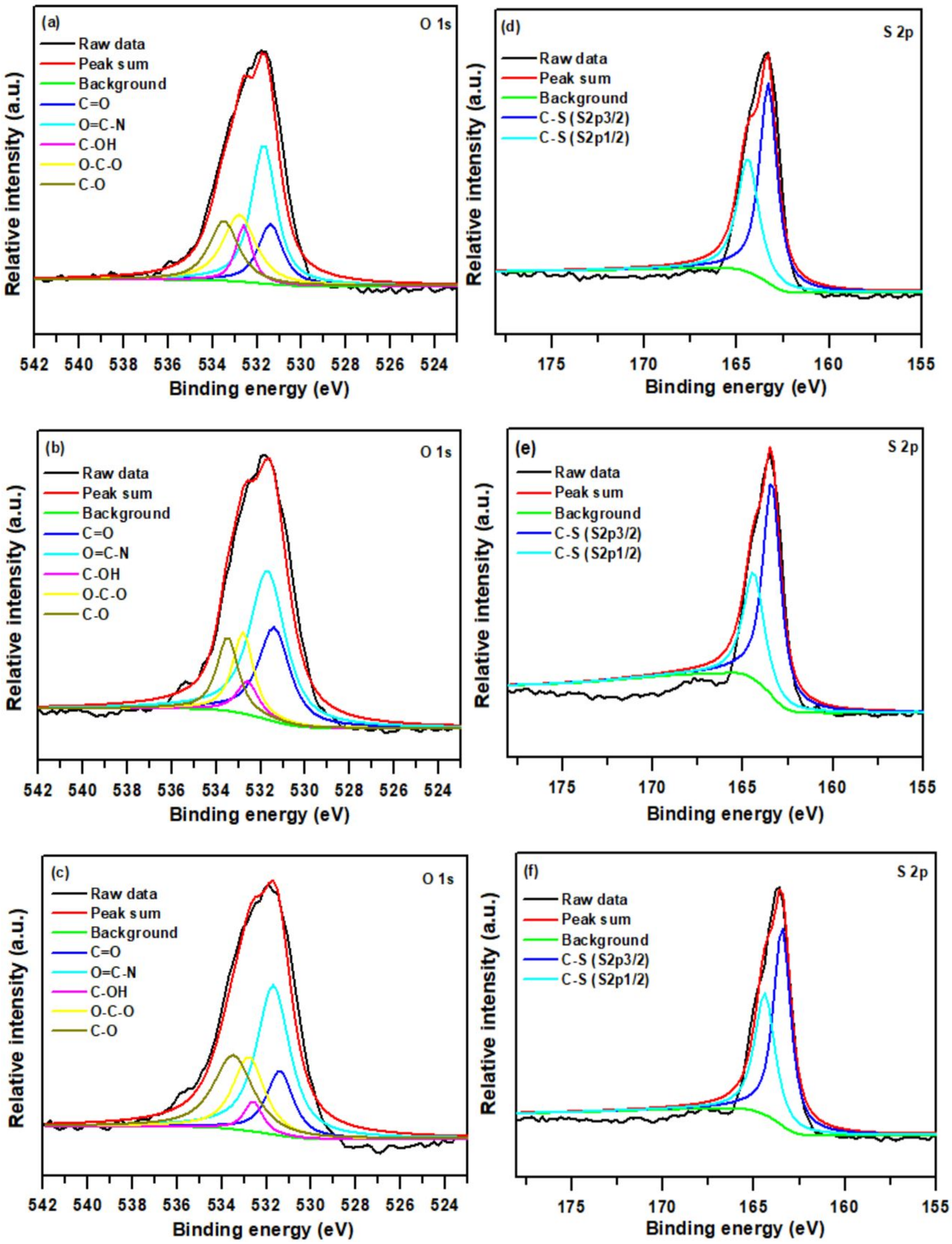


Figure S6. XPS spectra of O1s [(a) TPBT@rGO-30, (b) TPBT@rGO-40, and (c) TPBT@rGO-50] and S2p [(d) TPBT@rGO-30, (e) TPBT@rGO-40, and (f) TPBT@rGO-50].

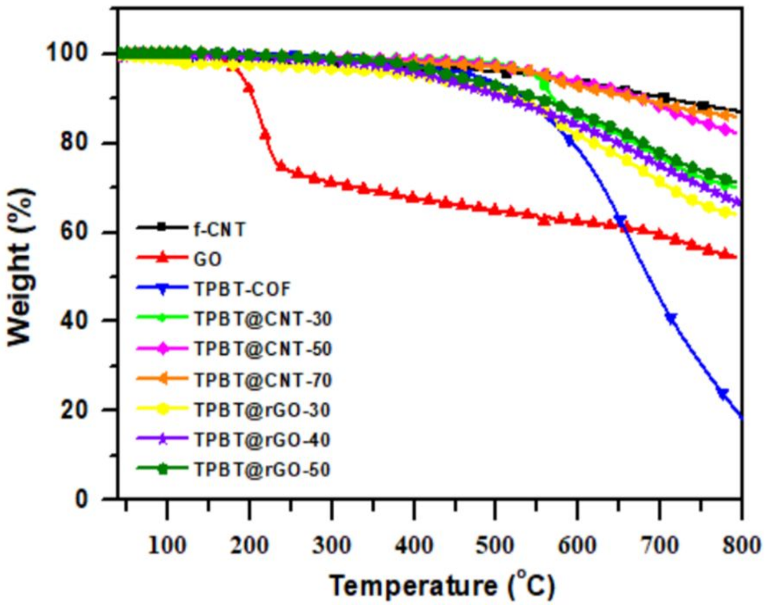


Figure S7. TGA thermograms of the TPBT@CNT and TPBT@rGO composite materials.

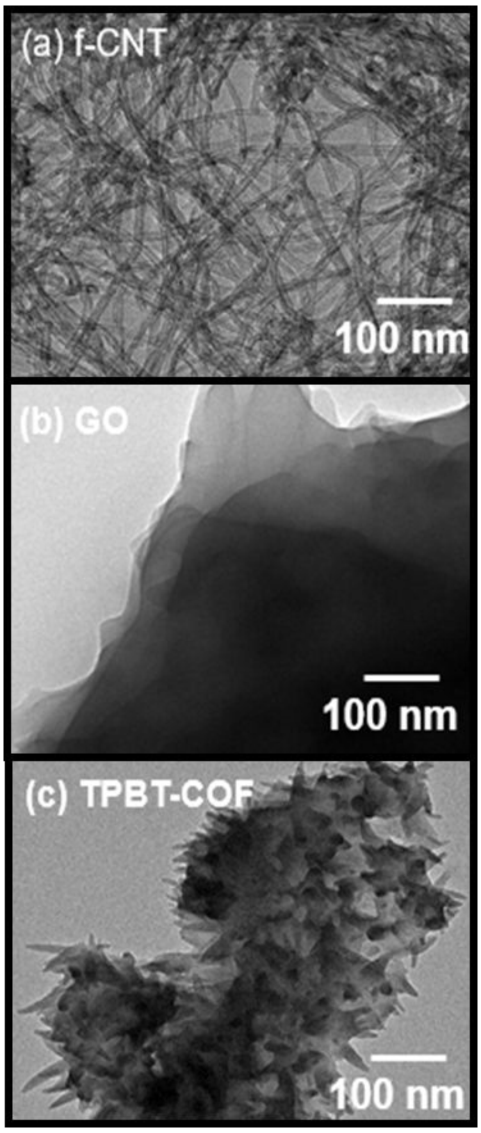


Figure S8. TEM images of (a) functionalized carbon nanotubes (f-CNT), (b) reduced graphene oxide (rGO), and (c) TPBT-COF.

Table S2. The specific surface areas and pore size of the TPBT@CNT and TPBT@rGO composites

| Composite | BET Surface Area ($m^2/g$) | Pore size (nm) |
|---|---|---|
| TPBT-COF | 1028.57 | 1.21 |
| TPBT@CNT-30 | 969.87 $m^2/g$ | 0.70 , 1.15 |
| TPBT@CNT-50 | 712.64 $m^2/g$ | 0.71 , 1.16 |
| TPBT@CNT-70 | 400.47 $m^2/g$ | 0.74 , 1.16 |
| TPBT@rGO-30 | 672.73 $m^2/g$ | 0.70 , 1.15 |
| TPBT@rGO-40 | 477.64 $m^2/g$ | 0.72 , 1.15 |
| TPBT@rGO-50 | 257.19 $m^2/g$ | 0.75 , 1.18 |

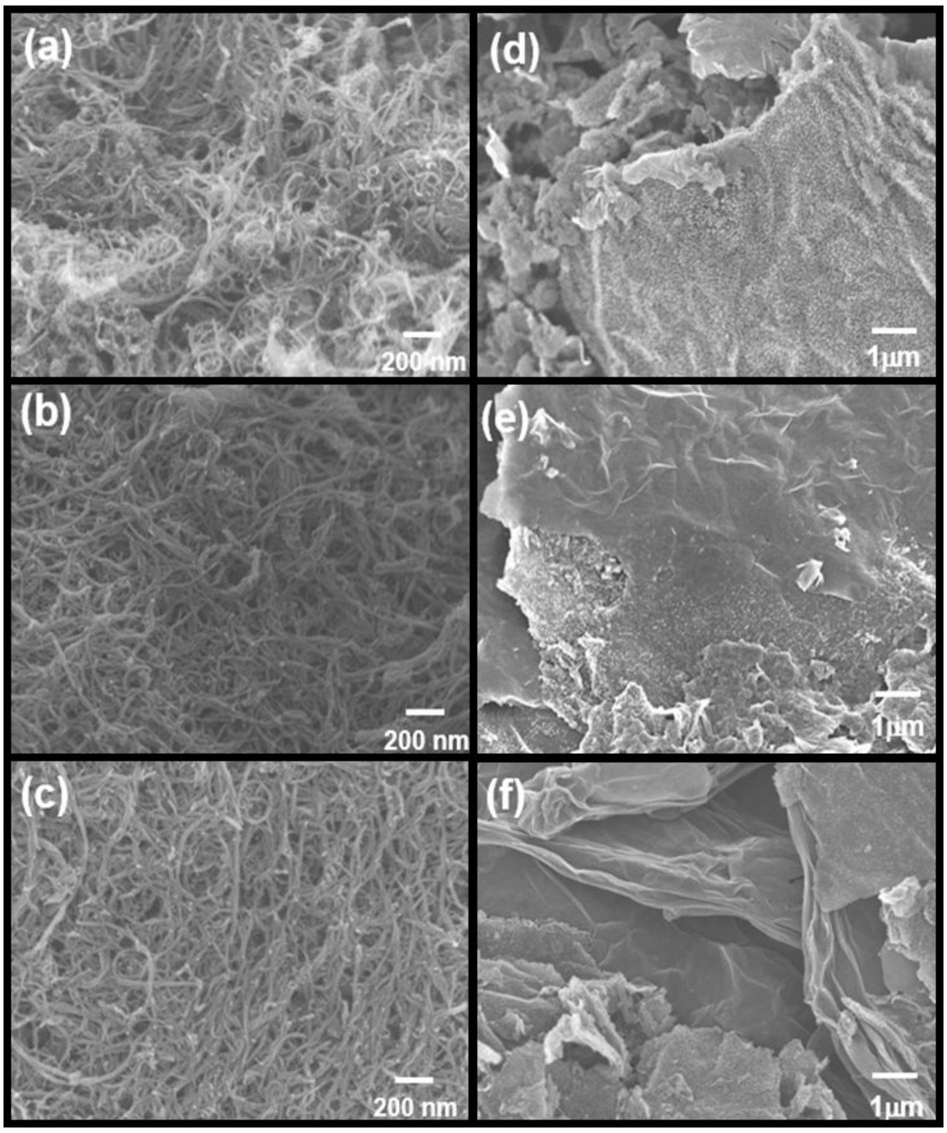


Figure S9 SEM images of composite films: (a) TPBT@CNT-30/CNT/RC (35/35/30, w/w), (b) TPBT@CNT-50/CNT/RC (35/35/30, w/w), (c) TPBT@CNT-70/CNT/RC (35/35/30, w/w), (d) TPBT@rGO-30/rGO/RC (35/35/30, w/w), (e) TPBT@rGO-40/rGO/RC (35/35/30, w/w), and (f) TPBT@rGO-50/rGO/RC (35/35/30, w/w).

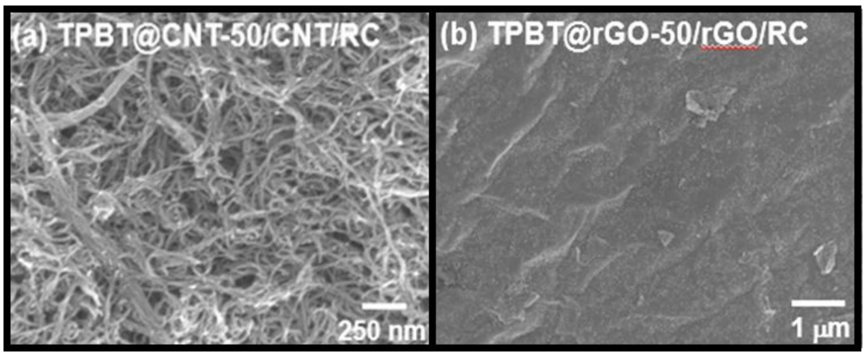


Figure S10 SEM images of the (a) CNT/RC (7:3, w/w) and (b) RGO/RC (7:3, w/w) composite films.

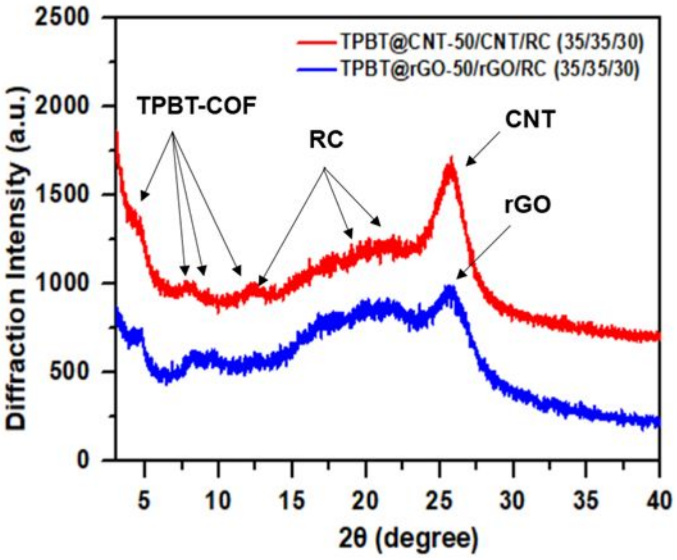


Figure S11 XRD images of the TPBT-COF@CNT/CNT/RC and TPBT-COF@rGO/rGO/RC composite films.

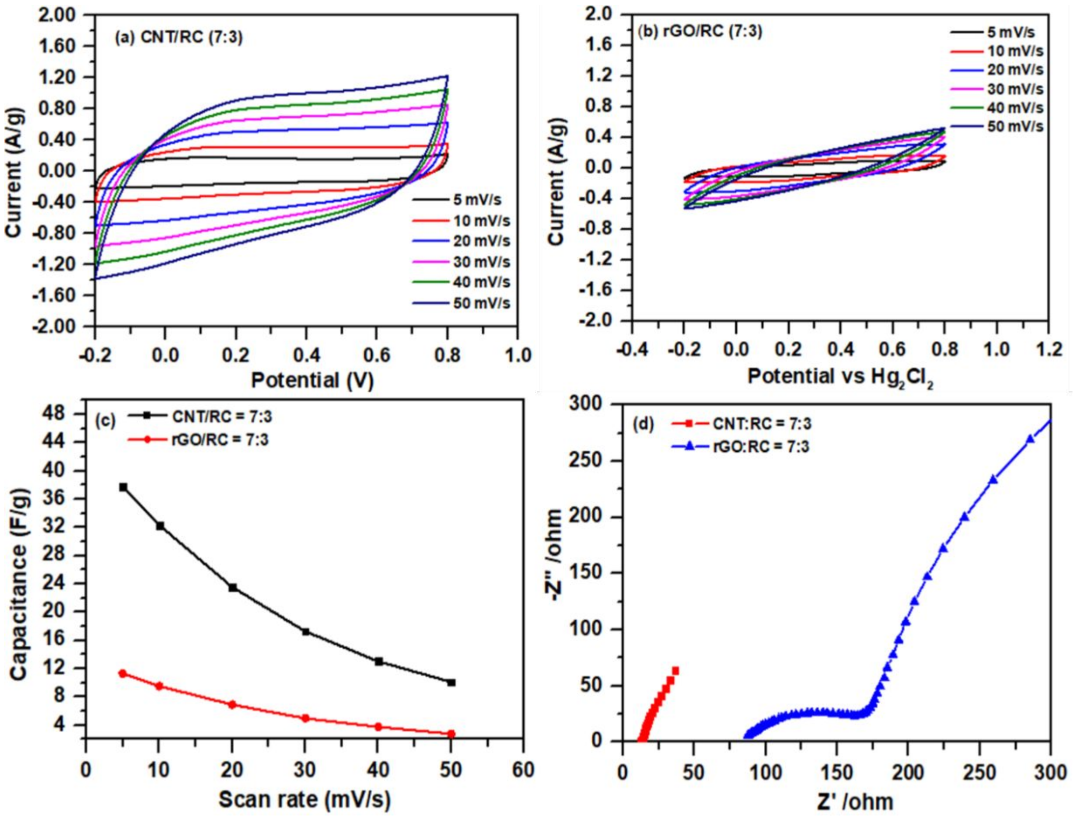


Figure S12. Electrochemical properties of CNT/RC (7:3, w/w) and rGO/RC (7:3, w/w) composite films: (a) CV curve of CNT/RC film, (b) CV curve of rGO/RC film, and (c,d) capacitance retention and EIS spectra of CNT/RC and rGO/RC films.

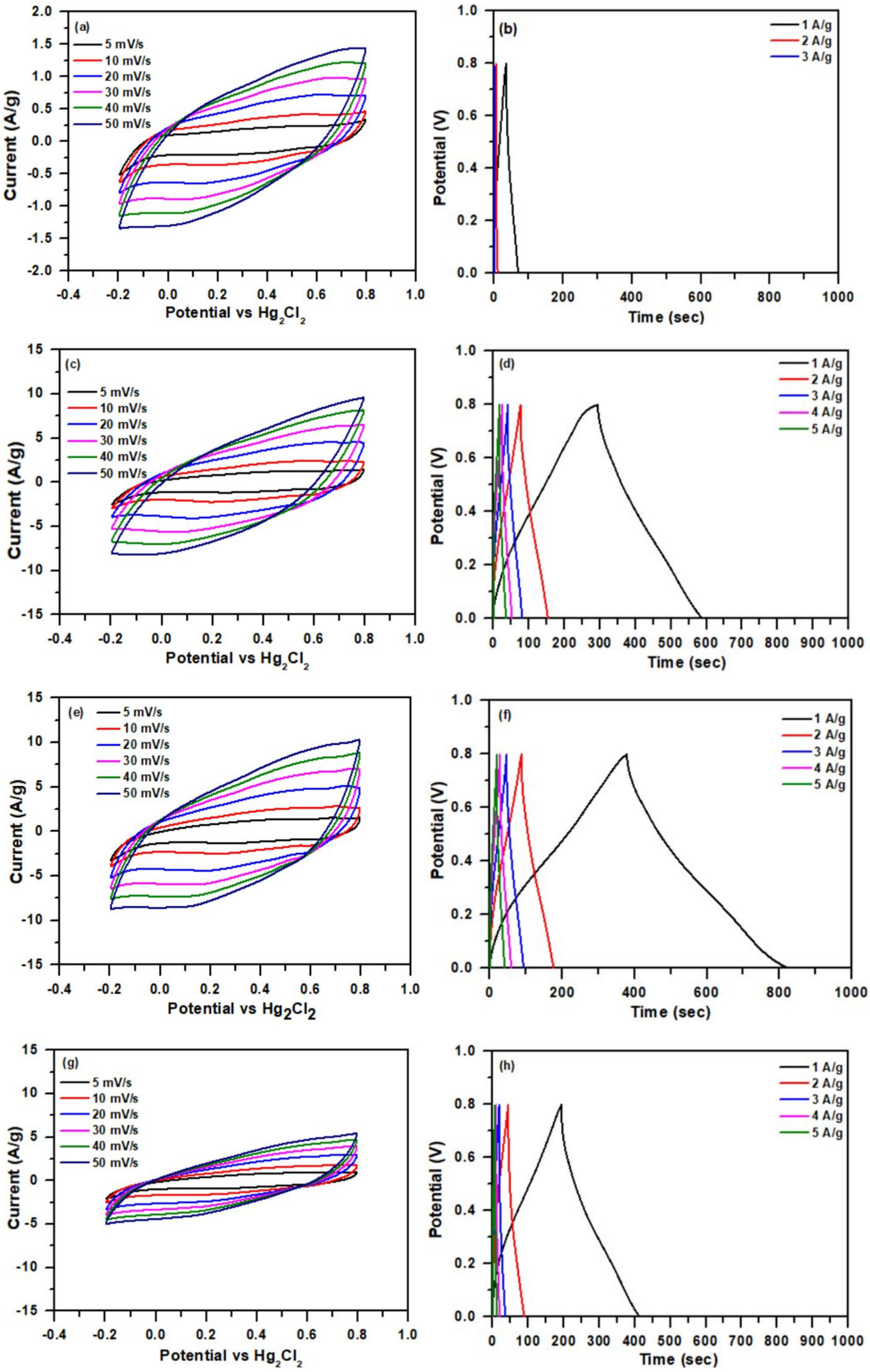


Figure S13 CV and GCD curves of composite films: (a,b) TPBT@CNT-50/CNT/RC (70/0/30, w/w), (c,d) TPBT@CNT-50/CNT/RC (46.7/23.3/30, w/w), (e,f) TPBT@CNT-50/CNT/RC (35/35/30, w/w), and (g,h) TPBT@CNT-50/CNT/RC (0/70/30, w/w).

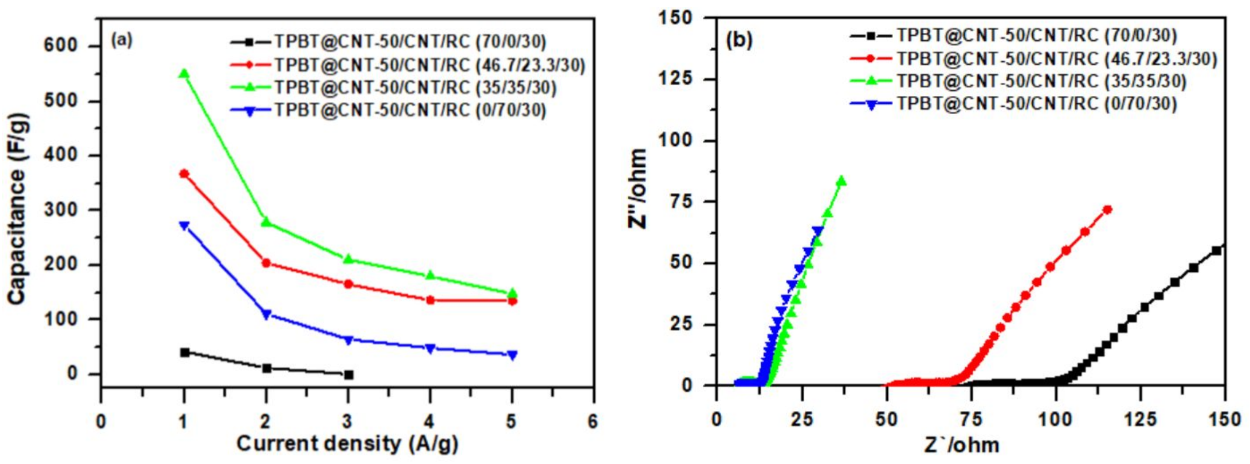


Figure S14 (a) Capacitance versus current density and (b) EIS diagrams of TPBT@CNT-50/CNT/RC composite films.

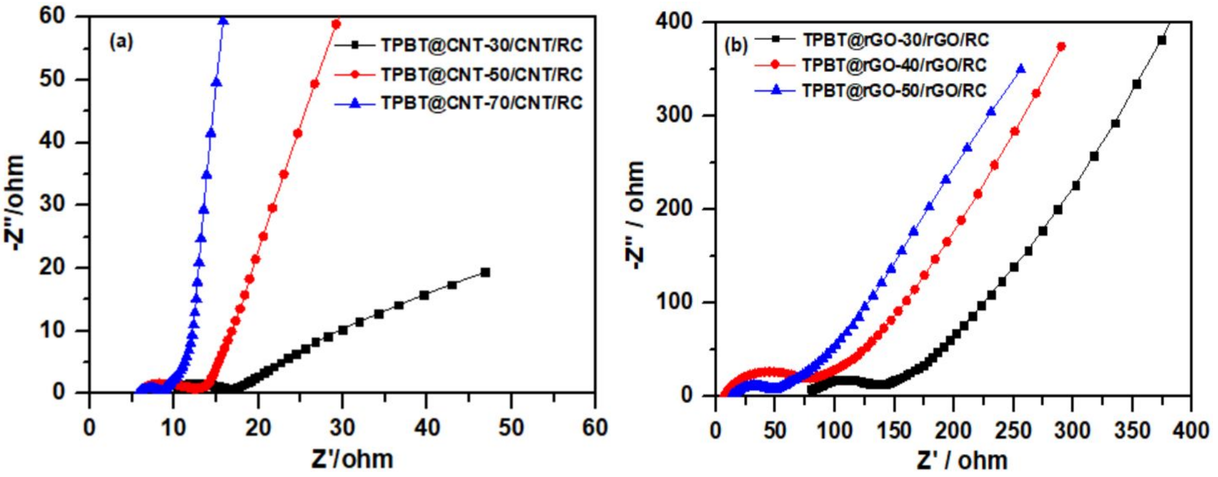


Figure S15. EIS diagrams of (a) TPBT@CNT/CNT/RC (35/35/30, w/w) and (b) TPBT@rGO/rGO/RC (35/35/30, w/w) composite films.

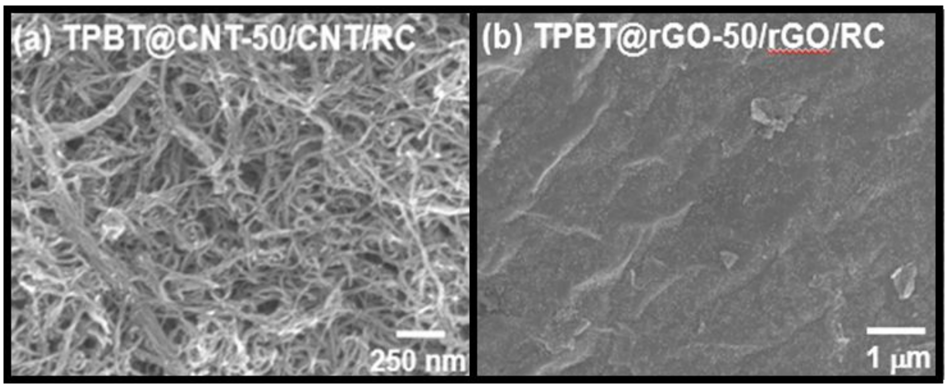


Figure S16. SEM images of the (a) PBT@CNT-50/CNT/RC (35/35/30, w/w) and (b) TPBT@rGO-50/rGO/RC (35/35/30, w/w) electrode after the 10,000 cycles test.

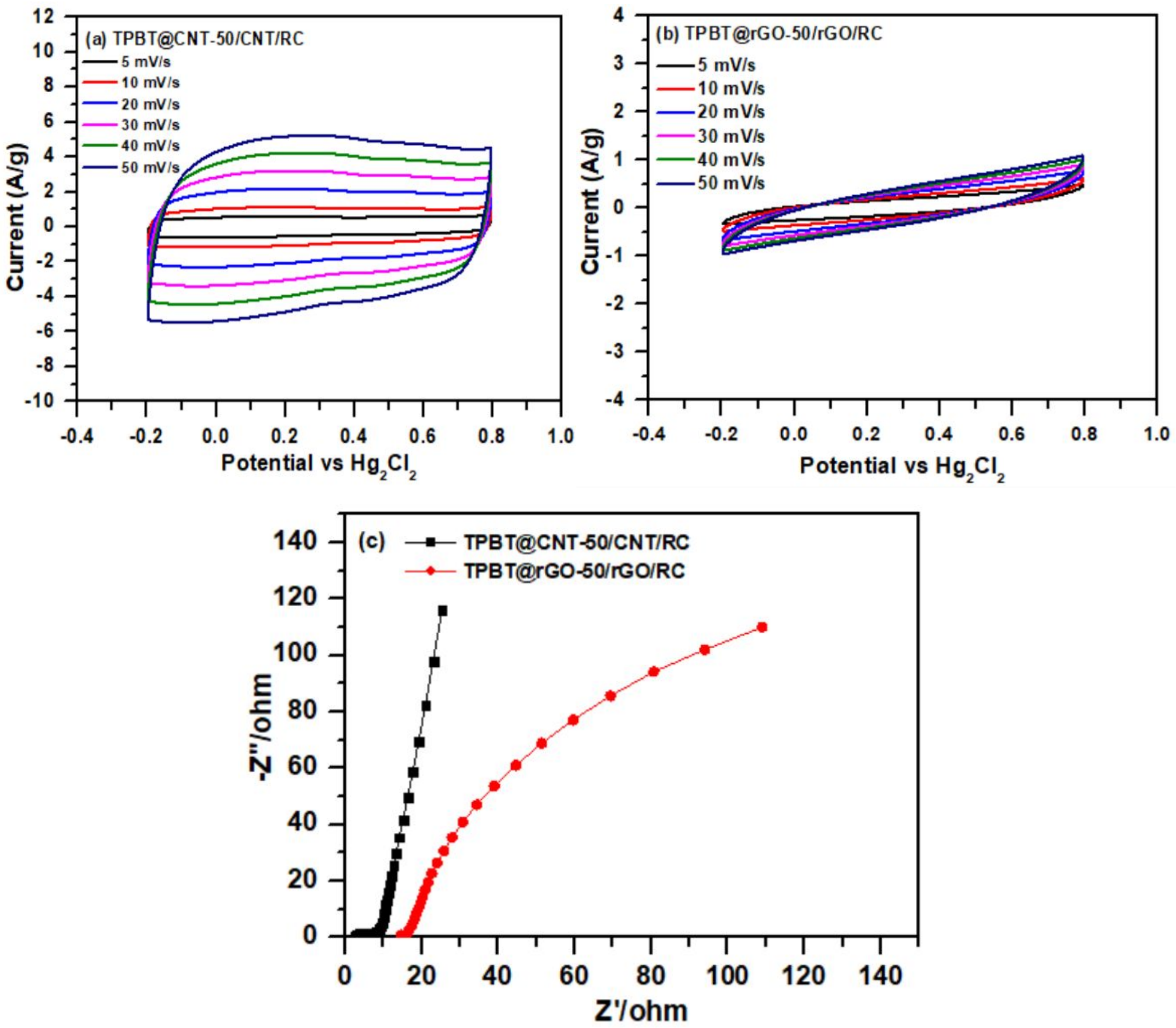


Figure S17. (a) CV curves of symmetric devices fabricated with TPBT@CNT-50/CNT/RC (35/35/30, w/w) films, (b) CV curves of symmetric devices fabricated with TPBT@rGO-50/rGO/RC (35/35/30, w/w) films, and (c) EIS diagram of the symmetric devices.

Table S3. Capacitance properties of triazine-based COF/carbon composite electrodes and their corresponding symmetrical supercapacitors.

| Electrodes | Electrolyte | Capacitance* (F/g) | Capacitance# (F/g) | Power# density (W/kg) | Energy# density (Wh/kg) | Cycling# test stability (%) | Ref. |
|---|---|---|---|---|---|---|---|
| TPBT@CNT/CNT/RC | 1M $H_2SO_4$ aqueous | 1288.3 | 84.3 | 312.5 | 11.7 | 77.0 after 10000 cycles | This study |
| TPBT@rGO/rGO/RC | 1M $H_2SO_4$ aqueous | 398.8 | 10.5 | 160.0 | 0.90 | 74.3 after 10000 cycles | This study |
| TPT-COF/graphite sheet | 0.5 M $K_2SO_4$ aqueous | 182.6 | 30.5 | 119.3 | 17.0 | 111.3 after 10000 cycles | 17 |
| CNT@TFA-COF-3 composite | 1M $H_2SO_4$ aqueous | 338.0 | -------- | ---- | ----- | 86.0 after 7000 cycles | 33 |
| TPTP-COF/f-CNT | 6M KOH aqueous | 577.4 | 56.4 | ----- | ----- | 78.6 after 10000 cycles | 35 |
| TPDA-COF/f-CNT | 6M KOH Aqueous | 640.4 | 70.6 | ----- | ----- | 81.5 after 10000 cycles | 35 |
| COF based N-doped carbon/rGO | 6M KOH aqueous | 234.0 | 161.8 | 400.0 | 14.3 | 86.0 after 3500 cycles | 36 |
| DMTA-TFP-COF/ graphite sheet | 1 M KCl aqueous | 434.0 | 61.5 | 320.2 | 12.2 | 99.3 after 5000 cycles | 51 |

* Specific capacitance of the triazine-COF/carbon composite electrode.

# Specific capacitance, power density, energy density, and cycle life stability of the triazine-COF/carbon composite electrode based symmetrical supercapacitor.
